# Creating healthy food environments in recreation and sport settings using choice architecture: a scoping review

**DOI:** 10.1093/heapro/daad098

**Published:** 2023-09-13

**Authors:** Rachel Prowse, Natasha Lawlor, Rachael Powell, Eva-Marie Neumann

**Affiliations:** Division of Community Health and Humanities, Faculty of Medicine, Memorial University of Newfoundland, 300 Prince Philip Drive, St. John's NL A1B 3V6, Canada; Division of Community Health and Humanities, Faculty of Medicine, Memorial University of Newfoundland, 300 Prince Philip Drive, St. John's NL A1B 3V6, Canada; Division of Community Health and Humanities, Faculty of Medicine, Memorial University of Newfoundland, 300 Prince Philip Drive, St. John's NL A1B 3V6, Canada; Library Services Division, Health Canada, Jeanne Mance Building, 200 Eglantine Driveway, Tunney’s Pasture, Ottawa, ON K1A 0K9, Canada

**Keywords:** nutrition, healthy settings, recreation, settings approach, sports facilities

## Abstract

Recreation and sport settings (RSS) are ideal for health promotion, however, they often promote unhealthy eating. Choice architecture, a strategy to nudge consumers towards healthier options, has not been comprehensively reviewed in RSS and indicators for setting-based multi-level, multi-component healthy eating interventions in RSS are lacking. This scoping review aimed to generate healthy food environment indicators for RSS by reviewing peer-reviewed and grey literature evidence mapped onto an adapted choice architecture framework. One hundred thirty-two documents were included in a systematic search after screening. Data were extracted and coded, first, according to Canada’s dietary guideline key messages, and were, second, mapped onto a choice architecture framework with eight nudging strategies (profile, portion, pricing, promotion, picks, priming, place and proximity) plus two multi-level factors (policy and people). We collated data to identify overarching guiding principles. We identified numerous indicators related to foods, water, sugary beverages, food marketing and sponsorship. There were four cross-cutting guiding principles: (i) healthy food and beverages are available, (ii) the pricing and placement of food and beverages favours healthy options, (iii) promotional messages related to food and beverages supports healthy eating and (iv) RSS are committed to supporting healthy eating and healthy food environments. The findings can be used to design nested, multipronged healthy food environment interventions. Future research is needed to test and systematically review the effectiveness of healthy eating interventions to identify the most promising indicators for setting-based health promotion in RSS.

**Contribution to Health Promotion**

- Recreation and sport settings can support healthy eating through carefully designed food environments.
- Based on health promotion principles, this scoping review developed a comprehensive list of indicators of healthy food environments for recreation and sport settings.
- These indicators can be mixed to create tailored, nested, multipronged interventions to support healthy eating in recreation and sport settings.
- Healthy food and beverage availability and marketing, reinforced through policies, are key components of healthy food environments in recreation and sport settings.

## INTRODUCTION

Setting-based interventions are accepted approaches to promote health in populations (World Health Organization (WHO), 2022; Poland *et al.*, 2009). Setting-based approaches embed health promotion in physical, organizational and social contexts in which health occurs and use multi-level interventions to comprehensively support health (Poland *et al.*, 2009). Settings range from microenvironments such as families and homes to large-scale settings such as workplaces, schools or cities (WHO, 2022). Recreation and sport settings (RSS), such as sports clubs and recreation facilities, are ideal settings for health promotion (Geidne *et al.*, 2013, 2019; Kokko *et al.*, 2014; Van Hoye *et al.*, 2021) due to their wide reach to children, educational focus, healthy living mandate and multi-level structure (Geidne *et al.*, 2019; Van Hoye *et al.*, 2021).

Setting-based interventions enable communities to move away from one-size-fits-all interventions to tailored, capacity-building interventions (Poland *et al.*, 2009; Kokko *et al.*, 2014). The success of implementing evidence-based interventions requires adapting to the context (Leeman *et al.*, 2017) thus tailored interventions may be particularly important for RSS in Canada, where these settings tend to be highly diverse with varied ownership and funding models, sport types, geography, food services and users. Naylor *et al*. created a framework of operational areas to support healthy eating in RSS which has guided experimental tailored interventions (Naylor *et al.*, 2010; Olstad *et al.*, 2019).

Some Canadian provinces developed voluntary provincial nutrition guidelines for RSS which had limited, but positive, impacts on healthy food availability in concessions and vending machines (Olstad *et al.*, 2020). In provinces with voluntary nutrition guidelines, food environments in RSS were further improved after an intensive, tailored 18-month capacity-building intervention that facilitated guideline implementation (Olstad *et al.*, 2019). Other research found, however, that this capacity-building intervention was unsuccessful at improving food marketing in the same facilities, presumably related to vague intervention goals (i.e. ‘market healthy choices’) set by intervention facilities (Prowse *et al.*, 2020). Applying principles of choice architecture to design environments to nudge consumers towards healthier options may improve the success of setting-based healthy eating interventions in RSS. Choice architecture has been studied in a variety of settings, including food retailers (Kraak *et al.*, 2017; Houghtaling *et al.*, 2019; Lindstrom *et al.*, 2022), workplaces (Al-Khudairy *et al.*, 2019; Rantala *et al.*, 2021), schools (Skov *et al.*, 2013; Nørnberg *et al.*, 2016) and health care settings (Al-Khudairy *et al.*, 2019), however, it has not been comprehensively reviewed in RSS.

Prowse *et al*. noted that the multiple levels that impact food environments in RSS, namely, individual factors (e.g. consumers), interpersonal factors (e.g. coaches and teams), institutional factors (e.g. food service providers, sports leagues,), community factors (e.g. sponsors, regional sport associations) and policy factors (provincial nutrition guidelines, municipal policies), were not collectively considered in the capacity-building intervention to improve food environments which may have curtailed the intervention’s impact (Prowse *et al.*, 2020). Given the diversity of RSS in Canada, it is important to consider the ‘added value’ of multi-level systems as a whole (Dooris, 2006). Multi-component and multi-level interventions in RSS are noted as promising interventions (Geidne *et al*., 2019), however, currently only broad theories and conceptual frameworks exist; indicators for setting-based multi-level/-component healthy eating interventions in RSS are lacking (Prowse *et al.*, 2020).

Active living, as well as other health-related behaviours such as tobacco and alcohol use (Geidne *et al.*, 2019; Van Hoye *et al.*, 2021), and more recently healthy eating (Pluim *et al.*, 2014; Wolfenden *et al.*, 2015; Westberg *et al.*, 2022), have been targets in health promotion interventions in RSS around the world. Setting-based approaches have been applied in RSS in Australia, Europe, Canada and the USA, however, few have focussed on nutrition (Geidne *et al.*, 2019). Guidance for setting-based health promotion initiatives for RSS have been developed, but multi-level setting-based healthy eating interventions are not common in the literature (Geidne *et al.*, 2019; Van Hoye *et al.*, 2021).

Canada recently released new national dietary guidelines which encourage public institutions, such as RSS, to adopt its key recommendations and support healthy food environments (Health Canada, 2022). To support the implementation of such broad health promotion recommendations, more information is needed for communities, practitioners and policy-makers to design, implement and evaluate interventions in RSS. We sought to identify indicators of healthy food environments, defined as measures of success described by goals, outcomes, indicators or targets, from peer-reviewed and grey literatures that can be used as a foundation to build tailored multi-level/-component interventions in RSS to promote healthy eating.

### Objective

This scoping review aimed to generate indicators of healthy food environments in RSS by reviewing interventions and outcomes in peer-reviewed and grey literature evidence. We aimed to explore indicators of healthy food environments based on principles of choice architecture (Kraak *et al.*, 2017) and multi-level setting-based health promotion approaches (Geidne *et al.*, 2019).

## METHOD

### Approach

We conducted a scoping review to systematically search and narratively describe existing evidence. Scoping reviews are valid approaches to literature reviews intending to provide an overview, clarify key concepts and identify key factors of a phenomenon (Munn *et al.*, 2018). This scoping review is reported based on the Preferred Reporting Items for Systematic Reviews and Meta-Analyses–Extension for Scoping Reviews (PRISMA-ScR) (Tricco *et al.*, 2018).

### Information sources and search strategy

A systematic search of peer-reviewed literature was conducted by a research librarian. The databases searched were Scopus, MEDLINE (Ovid), Embase (Ovid), Food Science and Technology Abstracts (Ovid), Global Health (Ovid) and APA PsycInfo (Ovid). Searches were conducted on 29 September 2021 and updated on 17 April 2023, restricted to articles published since 1 January 2011 and written in English or French. Minimal studies on food environment interventions in RSS exist before 2011. Detailed search strategies are in Supplementary File 1. The search results were uploaded into the RefWorks 2.0 (ProQuest LLC). Duplicate articles identified among databases were removed.

In September–October 2021, we searched Google for grey literature in English and French, such as existing nutrition guidelines, policies, practices, toolkits and other resources to support healthy eating and food environments in RSS. Searches strings were run with and without regional (Canada, Australia, New Zealand, USA and UK) and file type (‘pdf’) restrictions. These regions were previously identified as developing and evaluating healthy eating interventions in RSS (Geidne *et al.*, 2019). We conducted targeted hand searching of relevant government and organizational websites from Canada (*n* = 15), Australia (*n* = 12), the UK (*n* = 3) and the USA (*n* = 3) (Supplementary File 1).

### Selection process

Titles and abstracts of peer-reviewed articles were screened by N.L. and R.P. The first five pages of Google search results were screened by N.L. and R.P. The page limit was chosen *a priori* considering the Google Search ranking of most relevant results in the first pages, and to keep the number of results to review manageable (Godin *et al.*, 2015; Lefebvre *et al*., 2022).

Our selection strategy was informed by understanding indicators (i.e. measures of ‘success’) of healthy food interventions in RSS, where,

- ‘Healthy food interventions’ included any implemented or recommended change to food provision, promotion or sale;
- ‘RSS’ included all types of single sport and multi-sport centres as well as the events and programs that may take place in those centres (e.g. tournaments, camps and leagues); and
- ‘Success’ represents primary and secondary outcomes, or recommended targets or goals related to food environments, food policy, food purchases and dietary intake to measure change as a result of ‘healthy food interventions’

Documents were included if they had detailed quantitative or qualitative methods or results related to food environment characteristics, actions and supports that were considered goals, targets, outcomes or indicators of healthy eating or healthy food environments in RSS. Only resources produced in Canada, USA, Australia, New Zealand, UK and the European Union were included; the RSS across these regions are likely to have more established health promotion initiatives and be reasonably comparable. See Supplementary File 2 for detailed inclusion/exclusion criteria.

Full text of relevant resources was obtained for review. Twenty per cent of full-text articles were double screened, with discrepancies resolved by the first author. The research team met after double screening to discuss differences in screening outcomes. Research assistants were provided with further training on inclusion/exclusion criteria based on consensus after discussing screening results to improve consistency; the remaining resources were screened by a single research assistant.

### Data extraction and synthesis

Documents were classified as formal policies (i.e. official or endorsed practices), guidelines/recommendations (i.e. suggested, but not mandatory, implemented or evaluated policies or practices), toolkits (i.e. resources to help implement policies and practices) or real-life practices (i.e. pilots or experiments). Three steps were completed to extract and synthesize data, moving from individual pieces of data from single studies and documents towards general themes. First, data were extracted and coded by Canada’s national dietary guideline key messages. Data extracted included document type, country, as well as outcomes, indicators and targets (i.e. measures of success). It is common for policy guidance from higher jurisdictions (e.g. national) to be used to inform local action (e.g. provincial, municipal, institutional). Canada’s Food Guide is recommended to be implemented in public institutions, such as RSS, therefore, we used Canada’s Food Guide to guide data extraction to increase the policy relevance of the data and demonstrate how national policies can be operationalized at a local level. Each of the measures of success was coded according to key message topics of Canada’s Food Guide (Health Canada, 2022):

1. Make water your drink of choice.
2. Make it a habit to eat a variety of healthy foods each day.
3. Have plenty of vegetables and fruits.
4. Choose whole grains.
5. Eat protein foods.
6. Choose protein foods that come from plants more often.
7. Limit highly processed foods and beverages.
8. Be aware that food marketing can influence your choices.
9. Be mindful of your eating habits.
10. Cook more often.
11. Enjoy your food.
12. Eat meals with others.

Second, we collated extracted data for each key message into four sets: beverages (key messages #1,7), foods (key messages #2,3,4,5,6,7), marketing (key message #8) and social aspects (key messages #9,10,11,12).

Third, through a series of consensus-building meetings with research staff, we mapped extracted data in each set onto eight nudging strategies adapted from a multi-component evidence-based framework for generating healthy food environments by Kraak *et al.* (Kraak *et al*., 2017) (Table 1). The mix of nudging strategies to create a healthy food environment is often referred to as ‘choice architecture’ where settings are designed to nudge consumers towards a healthy option (Hollands *et al.*, 2013). Choice architecture has emerged in the evidence as a strategy to support healthy eating (Hollands *et al.*, 2013), and enhance the adoption of nutrition guidelines in populations (Enstaff, 2021). We added two new categories, Policy and People, to capture multi-level influences on healthy settings in recreation and sport (Kokko *et al.*, 2014). The adapted framework is henceforth referred to as the Healthy Eating Environments in Recreation and Sport Settings (HEERSS) Framework (Table 1). The data synthesized in the HEERSS Framework represent indicators for achieving healthy food environments in RSS which can be embedded into programs, practices or policies to promote healthy eating through multi-level/-component interventions. Finally, we categorized indicators that were collated within and between Canada’s Food Guide data sets and the HEERSS framework to develop general principles for action.

**Table 1:** Healthy eating environments in recreation and sport settings (HEERSS) framework

Component	Definition (adapted from Kraak *et al*., 2017)
Profile	The nutritional content, or healthfulness of foods and beverages provided, offered or sold in food outlets, through catering and in sports
Portion	The portion size of foods and beverages provided, offer or sold in food outlets, through catering and in sports
Pricing	The relative prices of foods and beverages sold in vending and concession. The provision of free foods or beverages at recreation and sport events, and around sport training and competition. Pricing strategies used to increase the value of foods and beverages.
Promotion	Commercial marketing practices to increase awareness, preference and choice of promoted foods and beverages in food outlets
Picks	Environmental changes to food outlets that normalize healthy default choices (e.g. salad as side)
Priming	Social marketing and healthy eating promotion undertaken by the facility, sport clubs, sports teams (coaches, players), staff and/or parents
Place	Changes to the facilities’ amenities to increase awareness of healthy food options (e.g. install water fountains or bottle filling stations)
Proximity	Placement of food and beverage options to increase the visibility of healthy options and decrease the visibility of less healthy options (e.g. placement of vending machines and water fountains, eye level of products).
Policy	Formalized written commitments to supporting healthy eating and food environments in recreation and sport facilities
People	Agents of change in recreation and sport facilities

Food outlets = vending and concession.

In sports = at recreation and sport events, and around sport training and competition.

## RESULTS

### Document characteristics

**Fig. 1:**
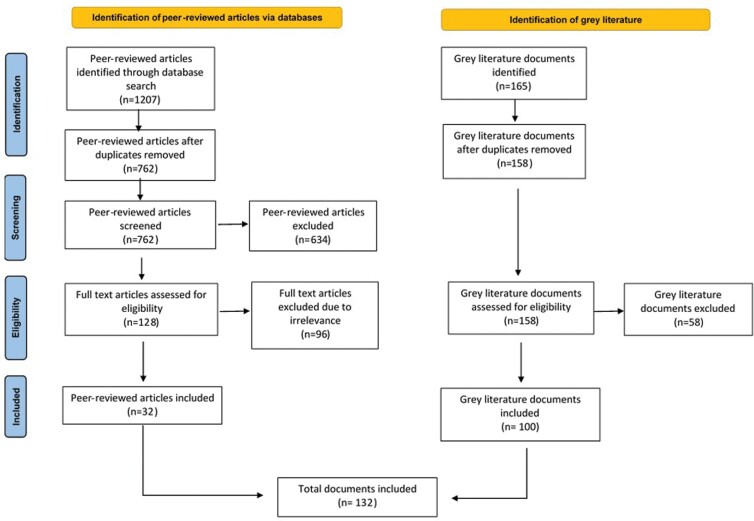
PRISMA diagram.

The systematic search resulted in 762 peer-reviewed articles, of which 128 were reviewed in full text; 165 grey literature resources were identified for full-text review. A total of 132 items were included, 100 grey documents (76%) and 32 peer-reviewed publications (24%), for data extraction (Figure 1).

Documents included guidelines/recommendations (*n* = 51, 39%), formal policies (*n* = 37, 28%), real-life practices (*n* = 33, 25%) and toolkits (*n* = 11, 8%). Most documents had general healthy eating recommendations (*n* = 68, 52%); however, fewer explicitly specified vegetables or fruit (*n* = 20, 15%), whole grains (*n* = 13, 10%) or protein foods (*n* = 10, 8%). Processed foods (*n* = 32, 25%) were slightly more commonly discussed. Water and sugary drinks were discussed in 35% (*n* = 46) and 28% (*n* = 37) of documents, respectively. Many had recommendations related to marketing (*n* = 87, 66%) or sponsorship (*n* = 41, 32%). Few (*n* = 12) discussed the social food environment. See Supplementary File 2 for the distribution of key message topics by reference identification (ID) number and document type.

We identified numerous indicators for healthy food and beverage environments which are summarized in Tables 2 and 3, respectively, organized by HEERSS component (see Supplementary File 2 for marketing data). We excluded the social environment from further analysis due to gaps in data using the HEERSS framework (see Supplementary File 2 for social environment data). From the indicators, we identified eight main indicator types, which fall into four guiding principles (Table 4). We review the data informing indicators for each guiding principle below.

**Table 2:** Indicators for Healthy Beverage Environments in Recreation and Sport Settings according to the HEERSS framework

HEERSS component	Indicators for water	Indicators for sugar-sweetened beverages (SSBs)
Profile	Water is available: Drinking, tap, potable, clean, safe water (4,10,23,30,33,39,49,54,55,64,65,66,67,76,77,80,82,85,89,94,103,105) Appealing/cool water (64,65,94,105) Plain/unflavoured water (33,76) Flavoured with no added sugar/caloric sweeteners (76,94) Unsweetened with no artificial sweeteners (83,97,103,123) or additives (caffeine, sodium) (83) Other beverages considered healthy or acceptable are sometimes available (depending on nutrient content and ingredients, and/or portion size): Milk (57,73,74,91,96,97,100,116) 100% Juice (57,74,91,93,96,97,100) Sports drinks (1, 109) Low calorie (e.g. <40 Kcal/Container) (33,57,70,96,97,132)	The following beverages are not available: Energy drinks (23,48,52,53,54,57,73,76,93,96,103) Soft drinks or soda (17,33,39,46,76,94,113,114) Fruit drinks (1,33,93,94,113) Caffeinated beverages (33,100) The following beverage may have limited availability: (see also ‘other beverages considered healthy or acceptable’): Flavoured milk (1,54,76,96,109,113) 100% juice (1,19,54,113,109) Flavoured water (76, 113) Sports drinks (33,39,53,54,94,113,132) Diet/artificially sweetened beverages (10,70,96,103,123)
Portion	No data	If available, SSBs are provided, offered or sold in smaller containers (e.g. smallest sell size) (23,33,39,93,76) ◦ 100% juice is limited to <4 oz (125 ml) to up to 12 oz (360 ml) (1,19,35,100) ◦ SSBs is limited to <200 ml or up to 355 ml (23,39) ◦ Flavoured milk is limited to <500 ml (76)
Pricing	Water is free (4,18,22,30,33,39,49,55,64,65,66,67,79,85,86,94,116) Bottle water is competitively priced (23,39,60,64,73,86,89,123) ◦ Bottled water cheaper than SSBs (23,39,63,73,123) Combo deals are used with water or healthy ­beverages (13,46,60,63,65,73,76,123)	Non-recommended beverages (e.g. SSBs) are more expensive than recommended beverages (e.g. water) (3,60,114) ◦ E.g. Soft drinks and fruit drinks cost $0.27/100 ml more than other beverages (114) Combo deals are not use with soft drinks (5)
Promotion	No data	SSBs are not promoted (57,87) See marketing for information on commercial marketing (e.g. sponsorship) with sugar-sweetened beverages
Picks	Water is available at all times (4,10,47,49,55,79,80,89,90,103) Water is promoted and provided as the preferred ‘drink of choice’ (2,12,77,92,97) Water and other healthy beverages make up at least 50% of available beverages (86,91,97,111,113,116) ◦ >50% (86,111,116) ◦ >70% (113) ◦ >75% (91) ◦ 100% (97; see also ‘SSBs are not available’) ◦ Increasing stocking of water (104) The variety of healthy beverage choices is increased (64,65) ◦ ≥2 types of water (or plain milk) available (116) ◦ 6 non-sugar beverages available (119)	SSBs are not available (1,17,47,74,109,111,113) or are only limitedly available (see ‘relative availability of water’) ◦ Less healthy beverages make up <20% of all beverages (60,111) ◦ Sports drinks make up <10% of all beverages (113) ◦ Decreased stocking of SSBs (104) Reduced variety of sugary drinks (e.g. Sports drinks, soft drinks) (46,64,65) Limit choice by offering fewer drinks items (93)
Priming	Visitors/athletes are encouraged to bring and use reusable water bottle (18,92,115,117) Coaches promote water before/during/after sports (92,116)	SSBs are rarely or never brought by children to sport activities/events (115)
Place	Water fountains (9,10,18,23,56,64,65,73,85,89,94,115) or other dispensers (e.g. bubblers, bottle filling) (4,14,39,49,66,67,79,117) are present	No data
Proximity	The visibility of water is increased (97,111,116) through: ◦ Access to water (e.g. fountains, vending machines with water) in playing areas (9,22,74) and high-traffic areas (49,64,65,66,67) ◦ Access to water at food outlets (22,46,79), catering (79) ◦ Relative availability of water (see Picks) ◦ Access to water at events (9,30,82,85) and in public spaces/buildings (9,85) ◦ Access to water for staff (4,33), visitors (4), players (22) and youth (33) ◦ Place water (healthy beverages) at eye level (39,46,60)	The visibility of less healthy beverages (e.g. sports drinks) is reduced by placing beverages behind the counter or out of sight (11,17,46,60,64,92,111) The opportunities to purchase and consume SSBs is reduced (105)
Policy	Mandated in policies: Water is promoted and provided as the preferred ‘drink of choice’ (2,77,92) Preference given to water, milk and 100% fruit juice (74, 91) Water always available (3,18,33,47,49,55,66,79, 80,82,89, 105) or offered (54) from water fountains (4,14,18, 49,66,79,89), food outlets (4,49,66,79), caterers (79) and in high-traffic areas (49, 66) Water always free (3,18,33,49,66, 79) or competitively priced (89) Water fountains are installed (14,18) in high-traffic areas (49,66) Encouraged to bring own water bottle (92)	Mandated in policies: No or limited sugary drinks (47, 54,74,105,109) ◦ Energy drinks (52,53, 54,96) ◦ Soft drinks (17,33) ◦ Juice drinks (33) ◦ 100% fruit juice (54) ◦ Sports drinks (33,53, 54,109) ◦ Chocolate (54) or sweetened milk (96,109) ◦ No caffeinated beverages (33) Limit 100% juice serving size (33,109)
People	Staff (1,5,47,109,114) Parents (2,117) Food service (canteen) (9,18,23) Food service (catering) (18,30,79) Coaches (22,116) Municipal councils/government ◦ Municipal councils (78) ◦ Regional government (13,14) Club committee members or representatives (22,91) Sports clubs (2,4,13,17,82,116,121) Sponsors (19)	

References are document identification numbers (ID) as per Table 1 in Supplementary File 2.

**Table 3:** Indicators for Healthy Food Environments in Recreation and Sport Settings according to the HEERSS framework

HEERSS component	Indicators for healthy foods (vegetables and fruit, whole grains, protein foods)	Indicators for highly processed foods
Profile	Healthy food is available (10,12,18,22,23,30,56,73,106) (see Picks) Foods are low in (added) fat, sugar and salt (7,10,19,34,38,45,52,57,90,94,95,96,97,100,103,104,126,130) Foods do not contain sugar substitutes/artificial sweeteners (103,123) Tiered ranking (nutrient profiling) system is used to classify foods according to healthfulness (4,7,10,11,13,18,19,23,25,38,40,45,46,48,49,52,55,61,64,66,67,76,79,81,82,83,89,90,92,93,94,95,101,103,106,112,118,124,126,127,129). Acceptability of foods sold and served depend on their ranking (e.g. Green/Most Healthy is more acceptable than Red/Least Healthy) Vegetables and fruit are available/offered (7,10,19,33,54,57,74,80,81,83,96,115,127) ◦ At every snack and meal (33,83) ◦ At most or all events (115) ◦ Daily (57) Whole grain foods are available/offered (7,10,19,33,54,57,63,83,96,100,104) Whole grains make up >50% of grain foods (7,19,33,47,50) ◦ >50% (7,19,47,50) ◦ 100% (33) Lean protein meats (7,10,19,33,47,57,80,101) and plant-based protein (7,10,19,33,47,57,80) are available/offered ◦ Always or regularly available (e.g. at every meal) (7,10,19,80) ◦ Only unprocessed cheese is available (7,19)	Unhealthy foods are not available (5,7,40,49,62,66,67,74,80,83,101,106) or are limitedly available (4,14,17,38,46,49,53,54,55,60,61,66,67,76,82,83,87,92,101,103,115,127) (see Picks) Processed meats are limited or ­avoided (83)
Portion	Serving size of foods is small (86) and meets dietary recommendations (e.g. CFG) (56,103) No supersized portions (55,96) Baked goods are sold in ‘moderate’ portions (7)	Serving size of unhealthy foods is reduced and/or sold in the smallest available single-serving size (10,13,22,49,56,57,63,64,66,67,81,89,93,96) ◦ E.g. <100 calorie/package snack (10) ◦ E.g. <200 calorie/serving food (100) No supersized portions (81,89)
Pricing	Healthy foods are competitively priced compared to unhealthy foods (10,13,19,23,24,39,45,55,56,57,73,76,81,82,90,93,94,96,100,116,119,121,128) ◦ e.g. Costs same or less (57,94), or is always cheaper (19,39,55,57,65,90,94,96,100) The price of healthy foods is decreased (13,23,34,45,64,91,92,96). Healthy foods are not charged a premium (10,56,96) Healthy foods are on sale through combos (13,20,24,64,65,76,83,87,91,116,123) or other promotions (e.g. BOGO, 50% off, loyalty programs) (13,20,64,65,76,94,128) Fruit is provided for free for facility visitors (e.g. Fruit bowl) (12) and given out to junior players (21) Prices are visibly displayed on menus (60,83)	The price of unhealthy foods is increased (13,23,53,63,64,65,83,92,96,114) to subsidize reduced prices of healthy foods (13,23,96) No combos with unhealthy foods (89) Unhealthy foods are not used for sale promotions (89)
Promotion	Healthy food is promoted (4,10,14,18,19,20,22,55,64,66,67,82,83,84,87,89,92,94,96,100,101, 104,122,123,128), through: ◦ Eye-catching signage, promotional signs menu boards (34,55,94,96,104,123) ◦ Colour and larger fonts (100,128) ◦ Presentation (plating, garnish and colour) (19,83,84) ◦ Attractive packaging (10,34,72,84) ◦ Listing healthy options at top of menu (39,57,72,84) ◦ Samples (23,24,84,128) Healthy eating posters are displayed (12,20,55,56,65,94,95,104,113,114,117,128) ◦ At point-of-purchase (65,104,128) ◦ Near eating/food areas (concessions, vending and eating areas) (19,22,94,110) ◦ On facility televisions (20) ◦ At entrances (114) Health promotion messaging must be of equal weighting to messages from food and sugary drink sponsors, if the latter exists (61) See marketing for information on commercial marketing (e.g. sponsorship) with healthy foods	Unhealthy foods and beverages promotion is restricted (4,13,14,27,28,29,30,35,36,37,42,43,44,49,59,60,64,65,66,67,79,82,87,89,101,103) No prominent promotion of unhealthy foods (4,13,14,49,60,64,65,66,67,79,87,89,101,103), including at ◦ Point-of-sale, cash registers (4,14) ◦ Near eating/food areas (concessions, vending and eating areas) (101) ◦ Reception desks, counters in waiting areas (4,14) ◦ Entrance/exits (4,14) ◦ On equipment (e.g. fridge) (4,13,14,60,101) Promotions for less healthy foods are not displayed at the expense of healthy foods (49,66,67,89) See marketing for information on commercial marketing (e.g. sponsorship) with unhealthy foods
Picks	Increased healthy food availability (11,12,21,23,43,45,63,64,71,87,105,110,118,) Healthy foods are available (% of all foods): ◦ 20% (93) ◦ ≥25% (56, 94) ◦ 30% (61%) ◦ 40% (93) ◦ ≥50% (4,7,14,17,18,32,34,38,46,49,55,60,61,66,67,71,76,79,81,87,89,90,93,95, 103,112,118,120 122) ◦ 70% (40) ◦ 80% (48,101) ◦ ≥85% (25) ◦ 90% (52) ◦ 100% (71) ▪ 100% of entrees are healthy (94,97) Increased variety of healthy foods (2,19,61,64,75,91,96,121) ◦ 5–6 healthy options (91,96) ◦ Increased stocking of healthier snacks (104) Plenty of vegetables and fruit (73) ◦ 1 fruit or vegetable ◦ 1–2 fruit options (57,80) ◦ 1 vegetable (57,80) ◦ 1 salad (80) ◦ at least 3 fruit or vegetables (116) At least 1 plant-based meal, 1 lean protein meal and 1 dairy (or plant-based) option (i.e. Yogurt or cheese) (81) White grain products are replaced with whole grain (7,19,63,104) Healthier sides are default in combo deals (20,34,101)	Decreased unhealthy food availability (43,45,62,73,80,110) Unhealthy foods are not or limitedly available (% of all foods): ◦ 0% (5,7,40,49,62,66,67,74,83,101,106) ◦ ≤10% (52,55,103,120,122) ◦ ≤15% (81) ◦ <20% (4,14,17,38,46,49,60,66,67,76,79,87,89,92,101,112) ◦ <50% (61) Reduced variety of unhealthy foods (64,93,96) ◦ Less than 2 options (96) Unhealthy foods are only sold a special events (106) Unhealthy sides are prohibited in combo deals (89) Limit choice by offering fewer menu items (93)
Priming	Club encourages parents and coaches to provide healthy snacks at sporting events, and for youth players (2,22,64,116,121) Club provides healthy snacks to youth players at sporting events (73,82,95) Traffic light schemes used to assess (4,13,14,18,20,25,38,45,46,49,61,64,66,67,76,79,82,87,89,92,101,106,112,118,129) and communicate (4,10,25,45,46,56,65,87,113,129) healthfulness to food services and consumers Other forms of menu/product labelling to highlight healthy options (10,19,56,84,100,114,131) Nutrition information is displayed for each product sold (57) Nutrition education is provided (5,22,64,65,78) See marketing for information on promotional posters, rewards/incentives and fundraising with healthy foods	See marketing for information on promotional posters, rewards/incentives and fundraising with unhealthy foods
Place	Food service equipment supports healthy food production and storage (e.g. Removal of deep-fryer (23,89,96); purchase of refrigerated vending machine (23))	
Proximity	Healthy food service options are closer to consumers than unhealthy food options (3) Vending machines with healthy choices are in ­high-traffic areas (83) Healthy food is promoted (4,10,13,14,18,19,20,22,49,52,55,64,66,67,82,83,84,87,89,92,94,96,100,101,104,117,123,128,132), including in prominent locations (4,10,13,14,18,19,33,49,66,67,67,82,89,101) Healthy foods are the most visible (5,6,1,11,13,23,24,34,40,56,57,60,63,64,72,76,83,84,91,92,94,113,114,123,132) ◦ At eye level (10,11,13,6,72,76,94,92,94,113,114,123,132), including children’s eye level (23) ▪ V/F placed at eye level (123) ◦ In highest selling potential positions (93) ◦ In priority locations: ▪ Point-of-sale, cash registers (13,23,73) ▪ Food service counters (23,55,93,124) ▪ Easy to access displays (72,84) ▪ Entrance/exits (13) ◦ Within top row (23,93), top third (57) and/or left-hand column (23,93), or right-hand column (93) of vending machine; or top and left-most buttons on vending machines (93) ◦ Within upper portion of fridge (91,123)	Unhealthy foods are not prominently placed (4,13,14,17,49,66,67,72,73,76,81,82,89, 93,101): ◦ Placed out of sight (13,64,73) ◦ Not at: ▪ Eye level on display (4,13,14) ▪ Point-of-sale, cash registers (4,13,14) ▪ Food service counters (64) ▪ Reception desks, counters in waiting areas (4,14) ▪ Entrance/ exits (4,13,14) ◦ In positions of lowest selling potential (93) Unhealthy food is not displayed in excessive quantities (101)
Policy	Mandates healthy food availability (2,4,5,6,15,18,23,32,49,52,55,66,71,75,77,78,79,80,81,89,91,92,95,98,99,105,108,113,115) and/or mandates reduced unhealthy food availability (82,98,99) ◦ Requires minimum and/or maximum of healthy and unhealthy food to be sold or offered, respectively (4,15,32,49,52,55,66,71,79,81,92,95,108) Implements tiered system (e.g. traffic lights) (4,5,6,14,18,32,45,49,52,55,66,79,81,82,89,92,95,108,123,124) Restrictions on food pricing (45,53,55,81,82, 89,91,113), placement (4,14,5,614,17,18,45,49,52,53,55,66,81,82,89,91,92,109) and promotion (4,14,16,18,49,52,55,66,79,82, 89,91,92,99,109,113) to favour healthy options Accreditation program exists with incentives (2,5,6,113) Change is implemented gradually (14,71,80) Health/recreation logo cannot be used to alongside unhealthy products (49,66,89)	
People	Staff (5,47,79) Parents (2,92) Food services (canteen) (9,18,23,40,48,49,93) Food services (vending) (23,32,40,49) Catered events/caterers (food service) (18,30,49) Coaches (22,92,116) Municipal councils/government ◦ Regional government (9,14,37,99,106) ◦ Municipal councils (78) Community groups (85) Club committee members or representatives (12,91) Sports clubs (2,82,116,121,122) Sponsors (19)	

References are document identification numbers (ID) as per Table 1 in Supplementary File 2.

**Table 4:** Guiding principles, indicators and potential actions to promote healthy eating and food environments in RSS

Guiding principle	Generic indicators	Potential actions
Healthy foods and beverages are available.	i. Healthy food and beverage availability is increased. ii. Unhealthy food and beverage availability is decreased.	Change absolute availability (e.g. present/absent) of healthy and unhealthy items Improve relative availability of healthy vs unhealthy items (e.g. proportion of menus, components of combo meals) Change variety of foods and beverages available Reduce portion sizes
The pricing and placement of food and beverages favours healthy options.	iii. Healthy food and beverages are competitively priced relative to unhealthy food. iv. Healthy food and beverages are more prominently (visibly) placed and accessible than unhealthy food and beverages.	Provide free water Improve relative affordability of healthy vs unhealthy items Change price promotions (e.g. combo deals, sales and loyalty discounts) Improve relative visibility of healthy vs unhealthy (e.g. eye level vs ‘out of sight’) Change proximity of healthy food access (e.g. vending) and options (e.g. point-of-purchase) to consumers
Promotional messages related to food and beverages supports healthy eating	v. Messages related to foods and beverages encourage healthy food and beverage practices. vi. Partnerships with businesses supports health rather than commercial motives.	Use social marketing to promote healthy foods (e.g. traffic light labelling) Restrict commercial unhealthy food marketing (e.g. sponsorship)
Facilities are committed to supporting healthy eating and healthy food environments.	vii. Healthy food availability and promotion are institutionalized through policies, contracts or other written documents. viii. The physical environment of facilities supports healthy food availability.	Develop and implement organizational or government policies Include healthy food requirements in food service contracts, leases, request for proposals, tenders Change facility infrastructure and equipment

### Guiding Principle 1: Healthy food and beverages are available

Overall, it was recommended that RSS increase the ­availability of healthy foods (document IDs: 11,12,21,23,43,45,63,64,71,87,105,110,118) and decrease the availability of less healthy foods (43,45,62,73,80,110). Many resources specified an acceptable minimum proportion of healthy foods and maximum proportion of unhealthy foods that should be available. The recommended relative availability of healthy foods ranged from 50% to 100% (4,7,14,17,18,25,32,34,38,40,46,48,49,52,55,60,61,66,67,71,76,79,81,87,89,90,93,95,101,103,112,118), while unhealthy food was commonly recommended to be >20% (4,14,17,38,46,49,52,55,60,66,67,76,79,81,87,89,92,101,103,112) or completely unavailable (5,7,40,49,62,66,67,74,83,101,106). Water (and other healthy beverages) were recommended to make up at least 50% of all beverages available (86,91,97,111,113,116); unacceptable beverages were recommended to be unavailable (1,17,47,74,109,111,113) or limitedly available (60,104,111,113). Progressively improving the relative availability of healthy foods through phased implementation of policies or guidelines was suggested (14,62,71,80,93,118).

In terms of specific food and beverage recommendations, the data suggested that free, plain, drinking water should be available to consumers at all times. Some resources also include other beverages as alternative healthy options, such as milk (57,73,74,91,96,97,116), 100% juice (57,74,91,93,96,97) and low calorie (<40 kcal) beverages (33,57,70,96,97,132). In general, energy drinks (23,48,52,53,54,57,73,76,93,96,103) (or any caffeinated beverages [33]), soft drinks or soda (17,33,39,46,76,94,113,114) and fruit drinks (1,33,93,94,113) were not recommended or allowed to be sold or served in RSS. Resources varied on their acceptability of sports drinks, with some allowing sports drinks (1,109), while others prohibited or limited sports drinks (33,39,53,54,94,113,132).

Vegetables and fruits were often mentioned that they should be available or offered in general (7,10,19,33,54,57,74,80,81,83,96,115,127). Whole grains were most often mentioned in regards to the proportion of grain products that should be whole, which ranged from 50% to 100% of grain products (7,19,33,47,50). Lean protein meats (7,10,19,33,47,57,80,101) and plant-based protein foods (7,10,19,33,47,57,80) were mentioned and were usually suggested to be ‘regularly’ available (7,10,19,80), although ‘regular’ was not defined. Processed meats were rarely mentioned specifically (83).

Food-related recommendations and policies were frequently based on a tiered nutrition profile ranking system (4,7,10,11,13,18,19,23,25,38,40,45,46,48,49,52,55,61,64,66,67,76,79,81,82,83,89,90,92,93,94,95,101,103,106,112,118,126,127,129), such as the Alberta Nutrition Guidelines for Children and Youth which uses ‘Choose Most Often’, ‘Choose Sometimes’ and ‘Choose Least Often’ (10). The most common type of tiered systems were green, yellow and red traffic light categories representing foods and beverages that are the healthiest, less healthy and least healthy, respectively (4,13,14,18,20,25,38,45,46,49,61,64,66,67,76,79,82,87,89,92,101,106,112,118,129). Various nutrition criteria (e.g. calories, nutrients and ingredients) were used as cut-offs to classify foods in tiers. Then, the tiers were incorporated into provision and promotion recommendations or policies. For example, the Victorian Healthy Choices policy in Australia requires that at least 50% of foods and beverages available be ‘green’ and no more than 20% of foods and beverages available be ‘red’ (112).

Smaller portion sizes were recommended for all food types (56,86,103); two documents recommended that portion sizes align with dietary recommendations (e.g. serving sizes from the 2007 Canada’s Food Guide [56,103]). Other resources stated that supersize portions were not acceptable (55,81,96,89). Reducing the serving size of unhealthy foods was most commonly recommended (10,13,22,49,56,57,63,64,66,67,81,89,93,96). Small portion sizes were recommended if sugar-sweetened beverages were offered (23,33,39,93,76).

### Guiding Principle 2: The pricing and placement of food and beverages favours healthy options

Water was recommended to be priced lower than sugar-sweetened beverages (23,60,89), and water was encouraged to be used in combo deals rather than sugar-sweetened beverages (5,60). Water was to be more visible than other beverages by placing it at eye level (60), offering it at food outlets (22,79) and by moving less healthy beverages out of sight (11,17,46,60,64,92).

Competitive pricing that favoured healthy food purchases over unhealthy foods was frequently mentioned (10,13,19,23,24,39,45,55,56,57,73,76,81,82,90,93,94,96,100,116,119,121,128). Of those that defined ‘competitive pricing’, 82% (*n* = 9) stated that healthy food should always be cheaper than unhealthy options (19,39,55,57,65,90,94,96,100); the remaining stated that healthy food should cost the same or less than unhealthy food (57,94). A few resources suggested that the price of unhealthy food should be increased to subsidized price reductions for healthy foods (13,23,96).

Numerous techniques to promote healthy food options were mentioned through attractive signage (34,55,94,96,100,104,123,128), strategic product placement (e.g. most visible [5,6,1,11,13, 23,24,34,40,56,57,60,63,64,72,76,83,84,91,92,94,113,114,123,132] or placed at eye level [10,11,13,6,72,76,94,92,94,113,114,123,132]), traffic light labelling (4,10,25,45,46,56,65,87,113,129) and healthy eating posters (12,20,55,56,65,94,95,104,113,114,117,128). Restricting unhealthy commercial food marketing was also mentioned (see Guiding Principle 3).

### Guiding Principle 3: Promotional messages related to food and beverages supports healthy eating

It was commonly recommended that unhealthy food and beverage marketing, in general, be restricted in sport or children’s settings (16,23,27,28,29,30,35,36,37,42,43,44,50,51,58,68,69,70,87,98,99,101,102,103,106,107,108). Many resources stated that unhealthy food marketing targeting children and youth specifically should be restricted (39,44,49,51,56,66,67,70,89,101,106,107,108). Although many resources stated that healthy foods should be promoted (4,10,13,14,18,19,20,22,49,52,55,64,66,67,82,83,84,87,89,92,94,96,100,101,104,117,123,128,132), only one resource stated that marketing to children should be limited to healthy foods (19). In some resources from Australia, it was stated as a condition of facility funding that all promotion, including sponsorship, related to unhealthy foods and beverages be restricted (16,27,42,43,50,69,98,99,102).

Sponsorship (16,21,22,26,27,31,37,41,42,42,49,50,53,66,67,69,89,98,99,102,106,107,108,116), fundraising (79,87,101,106,107,108) and rewards (15,79,87) (e.g. giveaways, prizes, incentives and vouchers) using unhealthy foods and beverages, or companies that represent the same, were discouraged or prohibited—particularly for children and youth sports and clubs. Healthy food and beverages, or non-food items were listed as acceptable options for sponsorship (14,19,55,64,73,76,77,91,92), fundraising (23) and rewards (76).

Social marketing activities were recommended to encourage water consumption including encouraging use of reusable water bottles (18,92,115,117) and promoting water consumption in children participating in sports and activities at the facility (2,12,77,92,97,116). Social marketing of healthy snacks, such as fruits and vegetables, by sports leagues, clubs and coaches were mentioned including the provision of free healthy snacks to youth players (73,82,95) and encouraging parents and coaches to bring healthy snacks to sporting events (2,22,64,116,121).

### Guiding Principle 4: Facilities are committed to supporting healthy eating and healthy food environments

Healthy food practices can be embedded in official policies by institutions, food service organizations (leases, contracts), municipal/regional governments and sport leagues, and can mandate indicators listed above. The majority of documents included real-life policies or practices (*n* = 70, 53%) (33+37) which suggest that these indicators are feasible to recommend and implement. Recommended policy actions for healthy food environments included mandated access to free water (3,18,33,49,66,79), restricted or limited sugar-sweetened beverage availability (47,54,74,105,109), increased healthy food availability (2,4,5,6,15,18,23,32,49,52,55,66,71,75,77,78,79,80,81,89,91,92,95,98,99,105,108,113,115), restricted food pricing, (45,53,55,81,82,89,91,113), placement (4,14,5,614,17,18,45,49,52,53,55,66,81,82,89,91,92,109) and promotion (4,14,16,18,49,52,55,66,79,82,89,91,92,99,109,113) to favour healthy options, and restricted sponsors in RSS (4,14,16,49,52,53,55,66,79,89,91,92,99,113).

The facility can also encourage water consumption by ensuring water fountains and bottle filling stations are present and easily accessible (4,9,10,14,18,23,39,49,56,64,65,66,67,73,79,85,89,94,115,117), or placed next to high-traffic areas (49,64,65,66,67) and playing areas (9,22).

## DISCUSSION

This review aimed to generate indicators of healthy food environments in RSS by summarizing peer-reviewed and grey literature on indicators of healthy eating and food environments in RSS. Numerous indicators related to foods, water and sugary beverages, as well as food marketing and sponsorship, were identified from the literature with a considerable amount from grey literature. We identified four principles for multi-level/-component healthy food environment interventions in RSS:

1. Healthy food and beverages are available.
2. The pricing and placement of food and beverages favours healthy options.
3. Promotional messages related to food and beverages supports healthy eating.
4. Facilities are committed to supporting healthy eating and healthy food environments.

Over the past decade, interventions to support healthy eating in RSS have focussed on three areas: guidelines/policy, organizational capacity-building and availability of healthy food and beverages (Westberg *et al.*, 2022). Guidelines/policy interventions have had limited impact without additional supports, such as capacity-building and tailored intervention planning (Westberg *et al.*, 2022). For example, recreation facilities in provinces with voluntary nutrition guidelines had significantly lower proportions of least healthy snacks and beverages compared to RSS in provinces without guidelines (76.5% vs 91.1% and 64.5% vs 80.8%, respectively). However, the majority of snacks and beverages were still considered unhealthy in both groups (Olstad *et al.*, 2020). Facilities that received a capacity-building intervention with tailored food environment changes further reduced the proportion of least healthy vending machine snacks by almost 30 percentage points, significantly greater than in the facilities that had nutrition guidelines but did not receive capacity-building support (Olstad *et al.*, 2019).

Implementing a variety of activities in RSS may mutually reinforce healthy eating practices. Nested, multipronged tailored interventions, supported by capacity-building are recommended as promising approaches to healthy food environments in RSS, which can be operationalized through a setting-based approach to health promotion (Von Hoye *et al.*, 2021). By collating together documents that relate to health promotion in RSS to identify indicators of healthy food environments that can be used to inform intervention design and evaluation, this review reinforces the promise of multi-level/-component interventions. By combining principles of choice architecture with settings approaches, it is possible that recreation and sport facilities and clubs may be able to more comprehensively promote health in their setting. Both choice architecture and setting-based approaches are known health promotion strategies (Hollands *et al.*, 2013; Geidne *et al.*, 2019); this is the first review to explicitly combine the two strategies in order to explore how food environments in RSS could be designed to support health.

Multi-level/-component setting-based approaches to healthy food environments may be more productive in creating and sustaining healthy food environments than singular interventions, regardless of if they are top-down or bottom-up. A limitation of provincial nutrition guidelines for RSS, and their use in capacity-building interventions, was that the guidelines were focussed on food and beverage availability and did not include many recommendations related to marketing (pricing, placement and promotion), or sponsorship (Prowse *et al.*, 2020), which could reinforce or oppose healthy food availability interventions.

Although this review does not identify the preferred mix of intervention indicators, our findings represent a menu of options from which a multi-level/-component intervention can be designed. Multi-level/-component interventions may help address real or perceived factors that influence healthy eating environments in RSS, including potential revenue loss of removing unhealthy food sponsors, lack of organizational capabilities and resources, and consumer preferences (Kirk *et al.*, 2021; Westberg *et al.*, 2022). Kirk *et al*. explain that barriers and facilitators of change to food environments in RSS are contextual and multi-level such as lack of champion for healthy eating environments (individual), staff support for initiative (interpersonal), capacity (organizational), demand for healthy options (community) and top-down mandatory guidelines (policy) (Kirk *et al.*, 2021). Progress towards healthy food environments in RSS is slowed due to the complexity of policy implementation, inadequate organizational support, social acceptability of unhealthy food in RSS, existing contracts with food service providers and sponsors and perceived financial challenges (Olstad *et al.*, 2011, 2012; Kirk *et al.*, 2021). Our findings, grounded in choice architecture and setting-based approaches to health promotion, can help change agents in RSS create tailored interventions to best suit their context, capitalize on opportunities and manage barriers.

Future research is needed to test and systematically review the effectiveness of healthy eating interventions in RSS to identify the most promising indicators, and strategies to achieve those indicators. Randomized controlled trials and natural experiments of food environment interventions for RSS are required to reduce the uncertainty of which types of interventions work best under which circumstances. Systematic reviews are required of indicators identified here (individually and in combination), such as the proportionate availability of healthy/unhealthy foods and beverages, traffic light labelling and restricting unhealthy food and beverage marketing, to identify best practices. Most of the evidence reviewed here was from grey literature, however, so it may be necessary to complete more primary research with common interventions and evaluation methods to facilitate a robust systematic review or meta-analysis. When underpinned by implementation science, experiments and evaluations of setting-based healthy food environment initiatives in RSS can provide valuable practice- and policy-relevant knowledge to adapt indicators to their context and create supportive physical, social, economic and political environments for healthy eating. Future research should explore how the social environment can support healthy eating in RSS—a gap identified here.

### Strengths and limitations

This scoping review included data from 132 peer-reviewed and grey literature resources. Since the literature search was limited to countries and years deemed most appropriate, there may be other relevant international or older research that was excluded. We searched the first five pages of Google search results; there may have been additional resources in later pages that would have been relevant. However, we found that resources were duplicated in later pages and between searches which suggests the grey search was comprehensive.

The review was informed by the components of the Canadian national guidelines, as well as literature on healthy food environments (Kraak *et al.*, 2017) and healthy settings in RSS (Kokko *et al.*, 2014; Geidne *et al.*, 2019). Using the choice architecture and multi-level influence frameworks to guide data extraction and interpretation increased the comprehensiveness and relevance of findings. Aligning data extraction with national dietary guidelines helped generate relevant indicators, enhances consistency in understanding and application of the guidelines and supports uptake of the guidelines by settings that influence diets at a population level.

Due to the volume and length of documents, data were extracted by a single research assistant which may have caused some errors or exclusion of relevant data. The peer-reviewed literature was screened and extracted by a single researcher. We were able to create a comprehensive list of indicators of healthy food environments in recreation and sport but did not assess the quality of the literature nor the effectiveness of individual indicators or mixes of indicators. Nonetheless, many documents included policies or practices that were recommended, trialled or implemented in real-life settings which enhances the applicability of the findings.

## CONCLUSIONS

This scoping review identified indicators of healthy food environments for RSS related to food and beverage availability and marketing from peer-reviewed and grey literature. The proposed indicators can be used as goals for RSS to design healthy eating interventions; they represent a collection of interventions that could be mixed and adapted to different contexts. Since improving food environments in RSS can be complex, challenging and slow, it may be important to allow flexibility in designing interventions to support healthy eating.

Local context impacts the effectiveness of public health interventions, thus the mix of interventions within a setting may be more important than the effectiveness of singular interventions. By exploring the ‘added value of the whole system approach’ (Dooris, 2006) in setting-based health promotion in RSS in future research, RSS may improve its contribution to health through healthy eating.

## Supplementary Material

daad098_suppl_Supplementary_File_S1

daad098_suppl_Supplementary_File_S2
