# Correction to: Creating healthy food environments in recreation and sport settings using choice architecture: a scoping review

**DOI:** 10.1093/heapro/daad178

**Published:** 2023-12-18

This is a correction to: Rachel Prowse, Natasha Lawlor, Rachael Powell, Eva-Marie Neumann, Creating healthy food environments in recreation and sport settings using choice architecture: a scoping review, *Health Promotion International*, Volume 38, Issue 5, October 2023, daad098, https://doi.org/10.1093/heapro/daad098.

In the originally published version of this manuscript, there was a typographical error in the author list.

This error has been corrected.

