# Supplementary material for: Creating healthy food environments in recreation and sport settings using choice architecture: a scoping review
Source: Health Promot Int. 2023 Sep 13;38(5):daad098. doi: 10.1093/heapro/daad098 (PMC10500220; doi:10.1093/heapro/daad098)
Supplement: daad098_suppl_Supplementary_File_S1 [file daad098_suppl_supplementary_file_s1.docx]

**Supplemental File 1**

Supplemental Tables 1 Search Strategies

Note: The original search strategies were limited to English. Searches were updated on April 17 2023, limited to English and to French. A search was also conducted to retrieve French language results for the original search period (1 January 2011 -September 29 2021).

Database(s): **Ovid MEDLINE(R) ALL**1946 to September 28, 2021
Search Strategy:

| **#** | **Searches** | **Results** |
| --- | --- | --- |
| 1 | "Sports and Recreational Facilities"/ | 100 |
| 2 | (bowling alley* or gym? or gymnasia or gymnasium or stadia? or stadium or swimming pool? or ((aquatic or athletic* or fitness or gymnastic? or leisure or multisport* or rec or recreati* or "recreation* and sport*" or sport* or ski or swim* or wellness) adj3 (center? or centre? or club? or complex* or facilities or facility)) or ((baseball or football or rugby or soccer or softball) adj3 (club? or event?)) or ((hockey or sport*) adj3 arena*) or ((recreation* or sport*) adj3 (environment? or event? or league* or organi?ation? or setting* or tournament* or venue? or youth?))).ti. | 3709 |
| 3 | (bowling alley* or gym? or gymnasia or gymnasium or stadia? or stadium or swimming pool? or ((aquatic or athletic* or fitness or gymnastic? or leisure or multisport* or rec or recreati* or "recreation* and sport*" or sport* or ski or swim* or wellness) adj3 (center? or centre? or club? or complex* or facilities or facility)) or ((baseball or football or rugby or soccer or softball) adj3 (club? or event?)) or ((hockey or sport*) adj3 arena*) or ((recreation* or sport*) adj3 (environment? or event? or league* or organi?ation? or setting* or tournament* or venue? or youth?))).ab. /freq=2 | 4136 |
| 4 | 1 or 2 or 3 [SPORTS SETTINGS] | 6448 |
| 5 | exp Beverages/ or Diet, Healthy/ or Food Services/ or exp Food/ or Food Preferences/ | 1421513 |
| 6 | (beverage* or diet? or dietary or eating? or food? or fruit? or meal? or nutrition* or snack? or vegetable* or (water adj2 (access or appeal* or ban or bans or bottle? or consum* or drink* or fountain* or intake* or plain or tap)) or cola? or soda? or SSB? or ((carbonat* or energy or nonalcoho?l* or "non alcoho?l*" or performance or power or soft or sugar* or sport? or sweet*) adj2 drink?)).ti. | 497473 |
| 7 | (beverage* or diet? or dietary or eating? or food? or fruit? o meal? or nutrition* or snack? or vegetable* or (water adj2 (access or appeal* or ban or bans or bottle? or consum* or drink* or fountain* or intake* or plain or tap)) or cola? or soda? or SSB? or ((carbonat* or energy or nonalcoho?l* or "non alcoho?l*" or performance or power or soft or sugar* or sport? or sweet*) adj2 drink?)).ab. /freq=2 | 594558 |
| 8 | (cafe or cafes or cafeteria? or canteen? or catering or concession? or foodservice* or menu? or vending or vendor?).tw,kw,kf. | 21935 |
| 9 | 5 or 6 or 7 or 8 [FOOD ENVIRONMENT] | 1929763 |
| 10 | capacity building/ or clinical trial/ or health promotion/ or nutrition policy/ or policy/ or exp guideline/ | 658411 |
| 11 | (campaign? or capacity building or checklist* or choice architecture or feasib* or guideline* or implement* or incentiv* or initiative* or intervention? or marketing or nudg* or policies or policy or (practice adj2 (best or promising)) or pricing or program? or programme* or promotion* or recommendation* or standard? or strateg* or toolkit? or tool kit? or transferab*).tw,kw,kf. | 5032364 |
| 12 | 10 or 11 [INTERVENTION] | 5487832 |
| 13 | 4 and 9 and 12 | 298 |
| 14 | limit 13 to (yr=2011-current and english) | 230 |

Database(s): **Embase**1974 to 2021 September 28
Search Strategy:

| **#** | **Searches** | **Results** |
| --- | --- | --- |
| 1 | exp sport facility/ | 2901 |
| 2 | (bowling alley* or gym? or gymnasia or gymnasium or stadia? or stadium or swimming pool? or ((aquatic or athletic* or fitness or gymnastic? or leisure or multisport* or rec or recreati* or "recreation* and sport*" or sport* or ski or swim* or wellness) adj3 (center? or centre? or club? or complex* or facilities or facility)) or ((baseball or football or rugby or soccer or softball) adj3 (club? or event?)) or ((hockey or sport*) adj3 arena*) or ((recreation* or sport*) adj3 (environment? or event? or league* or organi?ation? or setting* or tournament* or venue? or youth?))).ti. | 4179 |
| 3 | (bowling alley* or gym? or gymnasia or gymnasium or stadia? or stadium or swimming pool? or ((aquatic or athletic* or fitness or gymnastic? or leisure or multisport* or rec or recreati* or "recreation* and sport*" or sport* or ski or swim* or wellness) adj3 (center? or centre? or club? or complex* or facilities or facility)) or ((baseball or football or rugby or soccer or softball) adj3 (club? or event?)) or ((hockey or sport*) adj3 arena*) or ((recreation* or sport*) adj3 (environment? or event? or league* or organi?ation? or setting* or tournament* or venue? or youth?))).ab. /freq=2 | 5383 |
| 4 | 1 or 2 or 3 [SPORTS SETTINGS] | 9341 |
| 5 | exp *beverage/ or *catering service/ or exp *food/ or *food preference/ or *healthy diet/ | 506385 |
| 6 | (beverage* or diet? or dietary or eating? or food? or fruit? or meal? or nutrition* or snack? or vegetable* or (water adj2 (access or appeal* or ban or bans or bottle? or consum* or drink* or fountain* or intake* or plain or tap)) or cola? or soda? or SSB? or ((carbonat* or energy or nonalcoho?l* or "non alcoho?l*" or performance or power or soft or sugar* or sport? or sweet*) adj2 drink?)).ti. | 562724 |
| 7 | (beverage* or diet? or dietary or eating? or food? or fruit? o meal? or nutrition* or snack? or vegetable* or (water adj2 (access or appeal* or ban or bans or bottle? or consum* or drink* or fountain* or intake* or plain or tap)) or cola? or soda? or SSB? or ((carbonat* or energy or nonalcoho?l* or "non alcoho?l*" or performance or power or soft or sugar* or sport? or sweet*) adj2 drink?)).ab. /freq=2 | 762271 |
| 8 | (cafe or cafes or cafeteria? or canteen? or catering or concession? or foodservice* or menu? or vending or vendor?).tw,kw. | 30466 |
| 9 | 5 or 6 or 7 or 8 [FOOD ENVIRONMENT] | 1325131 |
| 10 | capacity building/ or clinical trial/ or health promotion/ or intervention study/ or nutrition policy/ or policy/ or practice guideline/ | 1691899 |
| 11 | (campaign? or capacity building or checklist* or choice architecture or feasib* or guideline* or implement* or incentiv* or initiative* or intervention? or marketing or nudg* or policies or policy or (practice adj2 (best or promising)) or pricing or program? or programme* or promotion* or recommendation* or standard? or strateg* or toolkit? or tool kit? or transferab*).tw,kw. | 6717194 |
| 12 | 10 or 11 [INTERVENTION] | 7654204 |
| 13 | 4 and 9 and 12 | 408 |
| 14 | limit 13 to (yr=2011-current and english) | 303 |

Database(s): **Global Health**1973 to 2021 Week 38
Search Strategy:

| **#** | **Searches** | **Results** |
| --- | --- | --- |
| 1 | health clubs/ or sports centres/ or recreational facilities/ | 875 |
| 2 | (bowling alley* or gym? or gymnasia or gymnasium or stadia? or stadium or swimming pool? or ((aquatic or athletic* or fitness or gymnastic? or leisure or multisport* or rec or recreati* or "recreation* and sport*" or sport* or ski or swim* or wellness) adj3 (center? or centre? or club? or complex* or facilities or facility)) or ((baseball or football or rugby or soccer or softball) adj3 (club? or event?)) or ((hockey or sport*) adj3 arena*) or ((recreation* or sport*) adj3 (environment? or event? or league* or organi?ation? or setting* or tournament* or venue? or youth?))).ti. | 1755 |
| 3 | (bowling alley* or gym? or gymnasia or gymnasium or stadia? or stadium or swimming pool? or ((aquatic or athletic* or fitness or gymnastic? or leisure or multisport* or rec or recreati* or "recreation* and sport*" or sport* or ski or swim* or wellness) adj3 (center? or centre? or club? or complex* or facilities or facility)) or ((baseball or football or rugby or soccer or softball) adj3 (club? or event?)) or ((hockey or sport*) adj3 arena*) or ((recreation* or sport*) adj3 (environment? or event? or league* or organi?ation? or setting* or tournament* or venue? or youth?))).ab. /freq=2 | 2290 |
| 4 | 1 or 2 or 3 [SPORTS SETTINGS] | 3508 |
| 5 | beverages/ or catering/ or exp food/ | 322578 |
| 6 | (beverage* or diet? or dietary or eating? or food? or fruit? or meal? or nutrition* or snack? or vegetable* or (water adj2 (access or appeal* or ban or bans or bottle? or consum* or drink* or fountain* or intake* or plain or tap)) or cola? or soda? or SSB? or ((carbonat* or energy or nonalcoho?l* or "non alcoho?l*" or performance or power or soft or sugar* or sport? or sweet*) adj2 drink?)).ti. | 372096 |
| 7 | (beverage* or diet? or dietary or eating? or food? or fruit? o meal? or nutrition* or snack? or vegetable* or (water adj2 (access or appeal* or ban or bans or bottle? or consum* or drink* or fountain* or intake* or plain or tap)) or cola? or soda? or SSB? or ((carbonat* or energy or nonalcoho?l* or "non alcoho?l*" or performance or power or soft or sugar* or sport? or sweet*) adj2 drink?)).ab. /freq=2 | 464083 |
| 8 | (cafe or cafes or cafeteria? or canteen? or catering or concession? or foodservice* or menu? or vending or vendor?).ti,ab. | 11841 |
| 9 | 5 or 6 or 7 or 8 [FOOD ENVIRONMENT] | 710921 |
| 10 | food policy/ or health promotion/ or guidelines/ | 86406 |
| 11 | (campaign? or capacity building or checklist* or choice architecture or feasib* or guideline* or implement* or incentiv* or initiative* or intervention? or marketing or nudg* or policies or policy or (practice adj2 (best or promising)) or pricing or program? or programme* or promotion* or recommendation* or standard? or strateg* or toolkit? or tool kit? or transferab*).ti,ab. | 947567 |
| 12 | 10 or 11 [INTERVENTION] | 953848 |
| 13 | 4 and 9 and 12 | 282 |
| 14 | limit 13 to (yr=2011-current and english) | 212 |

Database(s): **Food Science and Technology Abstracts**1969 to 2021 September Week 4
Search Strategy:

| **#** | **Searches** | **Results** |
| --- | --- | --- |
| 1 | (bowling alley* or gym? or gymnasia or gymnasium or stadia? or stadium or swimming pool? or ((aquatic or athletic* or fitness or gymnastic? or leisure or multisport* or rec or recreati* or "recreation* and sport*" or sport* or ski or swim* or wellness) adj3 (center? or centre? or club? or complex* or facilities or facility)) or ((baseball or football or rugby or soccer or softball) adj3 (club? or event?)) or ((hockey or sport*) adj3 arena*) or ((recreation* or sport*) adj3 (environment? or event? or league* or organi?ation? or setting* or tournament* or venue? or youth?))).ti. | 138 |
| 2 | (bowling alley* or gym? or gymnasia or gymnasium or stadia? or stadium or swimming pool? or ((aquatic or athletic* or fitness or gymnastic? or leisure or multisport* or rec or recreati* or "recreation* and sport*" or sport* or ski or swim* or wellness) adj3 (center? or centre? or club? or complex* or facilities or facility)) or ((baseball or football or rugby or soccer or softball) adj3 (club? or event?)) or ((hockey or sport*) adj3 arena*) or ((recreation* or sport*) adj3 (environment? or event? or league* or organi?ation? or setting* or tournament* or venue? or youth?))).ab. /freq=2 | 220 |
| 3 | 1 or 2 [SPORTS SETTINGS] | 271 |
| 4 | exp beverages/ or cafeterias/ or canteens/ or catering/ or catering establishments/ or foods service/ or exp foods/ or healthy eating/ or vending machines/ | 1288906 |
| 5 | (beverage* or diet? or dietary or eating? or food? or fruit? or meal? or nutrition* or snack? or vegetable* or (water adj2 (access or appeal* or ban or bans or bottle? or consum* or drink* or fountain* or intake* or plain or tap)) or cola? or soda? or SSB? or ((carbonat* or energy or nonalcoho?l* or "non alcoho?l*" or performance or power or soft or sugar* or sport? or sweet*) adj2 drink?)).ti. | 383129 |
| 6 | (beverage* or diet? or dietary or eating? or food? or fruit? o meal? or nutrition* or snack? or vegetable* or (water adj2 (access or appeal* or ban or bans or bottle? or consum* or drink* or fountain* or intake* or plain or tap)) or cola? or soda? or SSB? or ((carbonat* or energy or nonalcoho?l* or "non alcoho?l*" or performance or power or soft or sugar* or sport? or sweet*) adj2 drink?)).ab. /freq=2 | 407728 |
| 7 | (cafe or cafes or cafeteria? or canteen? or catering or concession? or foodservice* or menu? or vending or vendor?).tw. | 14995 |
| 8 | 4 or 5 or 6 or 7 [FOOD ENVIRONMENT] | 1433683 |
| 9 | exp food policy/ or guidelines/ or health promotion/ | 28387 |
| 10 | (campaign? or capacity building or checklist* or choice architecture or feasib* or guideline* or implement* or incentiv* or initiative* or intervention? or marketing or nudg* or policies or policy or (practice adj2 (best or promising)) or pricing or program? or programme* or promotion* or recommendation* or standard? or strateg* or toolkit? or tool kit? or transferab*).tw. | 306274 |
| 11 | 9 or 10 [INTERVENTION] | 308741 |
| 12 | 3 and 8 and 11 | 148 |
| 13 | limit 12 to (yr=2011-current and english) | 129 |

**"**

Database(s): **APA PsycInfo**1806 to September Week 3 2021
Search Strategy:

| **#** | **Searches** | **Results** |
| --- | --- | --- |
| 1 | (bowling alley* or gym? or gymnasia or gymnasium or stadia? or stadium or swimming pool? or ((aquatic or athletic* or fitness or gymnastic? or leisure or multisport* or rec or recreati* or "recreation* and sport*" or sport* or ski or swim* or wellness) adj3 (center? or centre? or club? or complex* or facilities or facility)) or ((baseball or football or rugby or soccer or softball) adj3 (club? or event?)) or ((hockey or sport*) adj3 arena*) or ((recreation* or sport*) adj3 (environment? or event? or league* or organi?ation? or setting* or tournament* or venue? or youth?))).ti. | 2230 |
| 2 | (bowling alley* or gym? or gymnasia or gymnasium or stadia? or stadium or swimming pool? or ((aquatic or athletic* or fitness or gymnastic? or leisure or multisport* or rec or recreati* or "recreation* and sport*" or sport* or ski or swim* or wellness) adj3 (center? or centre? or club? or complex* or facilities or facility)) or ((baseball or football or rugby or soccer or softball) adj3 (club? or event?)) or ((hockey or sport*) adj3 arena*) or ((recreation* or sport*) adj3 (environment? or event? or league* or organi?ation? or setting* or tournament* or venue? or youth?))).ab. /freq=2 | 2791 |
| 3 | 1 or 2 [SPORTS SETTINGS] | 3910 |
| 4 | exp "beverages (nonalcoholic)"/ or exp food/ or food preferences/ | 22088 |
| 5 | (beverage* or diet? or dietary or eating? or food? or fruit? or meal? or nutrition* or snack? or vegetable* or (water adj2 (access or appeal* or ban or bans or bottle? or consum* or drink* or fountain* or intake* or plain or tap)) or cola? or soda? or SSB? or ((carbonat* or energy or nonalcoho?l* or "non alcoho?l*" or performance or power or soft or sugar* or sport? or sweet*) adj2 drink?)).ti. | 62234 |
| 6 | (beverage* or diet? or dietary or eating? or food? or fruit? o meal? or nutrition* or snack? or vegetable* or (water adj2 (access or appeal* or ban or bans or bottle? or consum* or drink* or fountain* or intake* or plain or tap)) or cola? or soda? or SSB? or ((carbonat* or energy or nonalcoho?l* or "non alcoho?l*" or performance or power or soft or sugar* or sport? or sweet*) adj2 drink?)).ab. /freq=2 | 93522 |
| 7 | (cafe or cafes or cafeteria? or canteen? or catering or concession? or foodservice* or menu? or vending or vendor?).mp. | 7657 |
| 8 | 4 or 5 or 6 or 7 [FOOD ENVIRONMENT] | 115177 |
| 9 | health care policy/ or health promotion/ or intervention/ or policy making/ | 129323 |
| 10 | (campaign? or capacity building or checklist* or choice architecture or feasib* or guideline* or implement* or incentiv* or initiative* or intervention? or marketing or nudg* or policies or policy or (practice adj2 (best or promising)) or pricing or program? or programme* or promotion* or recommendation* or standard? or strateg* or toolkit? or tool kit? or transferab*).mp. | 1558345 |
| 11 | 9 or 10 [INTERVENTION] | 1558345 |
| 12 | 3 and 8 and 11 | 93 |
| 13 | limit 12 to (yr=2011-current and english) | 69 |

**SCOPUS**

**Search: September 29, 2021**

**Results: 264**

((TITLE-ABS("bowling alley" or gym or gyms or gymnasia or gymnasium or stadia or stadium or "swimming pool")) OR (TITLE-ABS((aquatic or athletic or fitness or gymnastic or leisure or multisport or rec or recreation or "recreation and sport" or sport or sports or ski or swim or wellness) W/3 (center or centre or club or complex or facilities or facility))) OR (TITLE-ABS((baseball or football or rugby or soccer or softball) W/3 (club or event))) OR (TITLE-ABS((hockey or sport) W/3 arena)) OR (TITLE-ABS((recreation or sport) W/3 (environment or event or league or organization or setting or tournament or venue or youth)))) AND ((TITLE(beverage or diet or dietary or eating or food or fruit or meal or nutrition or snack or vegetable)) OR (TITLE(water W/2 (access or appeal or ban or bans or bottle or consumption or drink or fountain or intake or plain or tap))) OR (TITLE(cola? or soda?)) OR (TITLE((carbonated or energy or nonalcohol or "non alcohol" or performance or power or soft or sugar or sport or sweetened) W/2 drink)) OR (TITLE(cafe or cafes or cafeteria or canteen or catering or concession or foodservice or menu or vending or vendor))) AND (TITLE-ABS(campaign or "capacity building" or checklist or "choice architecture" or feasible or feasibility or guideline or implementation or incentive or initiative or intervention or marketing or nudge or nudging or policies or policy or "best practice" or "promising practice" or pricing or program or programme or promotion or recommendation or standard or strategies or strategy or toolkit or "tool kit" or transferable)) AND ( LIMIT-TO ( PUBYEAR,2021) OR LIMIT-TO ( PUBYEAR,2020) OR LIMIT-TO ( PUBYEAR,2019) OR LIMIT-TO ( PUBYEAR,2018) OR LIMIT-TO ( PUBYEAR,2017) OR LIMIT-TO ( PUBYEAR,2016) OR LIMIT-TO ( PUBYEAR,2015) OR LIMIT-TO ( PUBYEAR,2014) OR LIMIT-TO ( PUBYEAR,2013) OR LIMIT-TO ( PUBYEAR,2012) OR LIMIT-TO ( PUBYEAR,2011) ) AND ( LIMIT-TO ( LANGUAGE,"English" ) )

**Supplemental Tables 2 – Grey Literature Searches**

**Google Search Strings**

**Site:** Google Search: <https://www.google.com/>

**Site**: Google Advanced Search: <https://www.google.ca/advanced_search>

The first 5 pages of results were reviewed for each search string.

| **Search Strategy** |
| --- |
| “healthy food” arena |
| “healthy food” “community sports” |
| “healthy food” “fitness centre” |
| “healthy food” gymnasium |
| “healthy food” “leisure centre” |
| “healthy food” “recreation |
| “healthy food” “recreation centre“ |
| “healthy food” “rec centre” |
| “healthy food” “recreation facility” |
| “healthy food” “recreation and sports” |
| “healthy food” “sports centre” |
| “healthy food” “sports club” |
| “healthy food” “sporting event” |
| “healthy food” “sports complex” |
| “healthy food” “sports facility” |
| “healthy food” “sports setting” |
| “healthy food” stadium |
| “healthy choices” “sports centre” |
| “nourriture saine” aréna |
| “nourriture saine” “sports communautaires” |
| “nourriture saine” “centre de fitness” |
| “nourriture saine” gymnase |
| “nourriture saine” “centre de loisirs” |
| “nourriture saine” “centre récréatif” |
| “nourriture saine” “centre de réc” |
| “nourriture saine” “recreation et sports” |
| “nourriture saine” “centre sportif” |
| “nourriture saine” “club sportif” |
| “nourriture saine” “événement sportif” |
| “nourriture saine” “complexe sportif” |
| “nourriture saine” stade |
| “nourriture saine” “centre sportif” |
| “choix sains” “centre sportif” |

**Websites Searched**

| **Website Title** | **Link** |
| --- | --- |
| Alberta Health Services | <https://albertahealthservices.ca/> |
| Alberta Health Services | <http://www.healthyeatingstartshere.ca/> |
| BC Interiror Health | <https://www.interiorhealth.ca/Pages/default.aspx> |
| Government of British Columbia, Health | <https://www2.gov.bc.ca/gov/content/health> |
| Government of New Brunswick (Public Health) | <https://www2.gnb.ca/content/gnb/en/departments/health.html> |
| Government of New Brunswick (Public Health) | <https://www2.gnb.ca/content/gnb/en/departments/ocmoh.html> |
| Healthy Eating Nova Scotia | <https://novascotia.ca/dhw/healthy-communities/healthy-eating.asp> |
| Institut national de sante publique du Québec (INSPQ) | <https://www.inspq.qc.ca/> |
| Alberta Policy Coalition for Chronic Disease Prevention (APCCP) | <https://abpolicycoalitionforprevention.ca/> |
| Alliance for Healthy Eating and Physical Activity (NS) | <http://www.nsalliance.ca/> |
| Dietitians of Canada | <https://www.dietitians.ca/> |
| Nutrition Connections | <https://nutritionconnections.ca/> |
| Ontario Dietitians in Public Health (previously Ontario Society of Nutrition Professionals in Public Health) | <https://www.odph.ca/> |
| Ontario Dietitians in Public Health (previously Ontario Society of Nutrition Professionals in Public Health) | <https://www.odph.ca/healthy-eating-in-the-recreation-setting> |
| Ontario Public Health Association | <https://opha.on.ca/> |
| Recreation Facility Association of Nova Scotia | <http://www.rfans.com/> |
| Recreation NL [Newfoundland & Labrador] Eat Great and Participate | <http://www.recreationnl.com/programs-events/eat-great-participate/> |
| Saskatchewan Parks and Recreation Association (SPRA) | <https://www.spra.sk.ca/> |
| Stay Active, Eat Healthy | <https://stayactiveeathealthy.ca/> |
| Australian Prevention Partnership Centre | <https://preventioncentre.org.au/> |
| Food Policy Index | <https://www.foodpolicyindex.org.au/> |
| Nutrition Australia Foundation | <https://nutritionaustralia.org/> |
| Government of Western Australia, Department of Health | <https://ww2.health.wa.gov.au/Home> |
| Queensland Government, Recreation, Sports and Arts | <https://www.qld.gov.au/recreation> |
| VicHealth | <https://www.vichealth.vic.gov.au/> |
| Victoria State Government | <https://www.vic.gov.au/> |
| Victoria State Government, Health Policies and guidelines | <https://www2.health.vic.gov.au/about/publications/policiesandguidelines> |
| Victoria State Government. Healthy Eating Advisory Service (HEAS) | <https://heas.health.vic.gov.au/> |
| Victoria State Government. Sport and Recreation Victoria | <https://sport.vic.gov.au/> |
| VicSport | <https://www.vicsport.com.au/> |
| Victoria State Government, Health | <https://www2.health.vic.gov.au/> |
| Gov.UK Healthy Eating | <https://www.gov.uk/health-and-social-care/healthy-eating> |
| Public Health England | <https://www.gov.uk/government/organisations/public-health-england> |
| Bridging the Gap | [http://www.bridgingthegapresearch.org](http://www.bridgingthegapresearch.org/) |
| Healthy Eating Research (national program of the Robert Wood Johnson Foundation) | <https://healthyeatingresearch.org/> |
| National Alliance for Nutrition and Activity | <https://cspinet.org/protecting-our-health/nutrition/national-alliance-nutrition-and-activity> |
