# Supplementary material for: Creating healthy food environments in recreation and sport settings using choice architecture: a scoping review
Source: Health Promot Int. 2023 Sep 13;38(5):daad098. doi: 10.1093/heapro/daad098 (PMC10500220; doi:10.1093/heapro/daad098)
Supplement: daad098_suppl_Supplementary_File_S2 [file daad098_suppl_supplementary_file_s2.docx]

**Supplemental File 2**

**Supplemental Table 1:** Inclusion and exclusion criteria

|  | Inclusion Criteria | Exclusion Criteria |
| --- | --- | --- |
| Topic | - Population-based healthy eating promotion or intervention (e.g. healthy food availability, concessions and vending, team nutrition) in recreation and sport settings (including school-based sport events) - Institutional (i.e. recreation facility) and/or retailer food environments | - Population-based healthy eating promotion or intervention in other settings - Non-population based interventions (e.g. personalized nutrition, individual sports nutrition, eating disorders) - Community food environments (i.e. geographic access to food retailers) |
| Outcomes | - Clear recommendations or indicators (i.e. ‘measures of success’) of healthy eating and food environments | - General or non-specific recommendations or indictors of healthy eating and food environments - Weight or obesity outcomes - Alcohol outcomes |
| Country | - Canada, Australia, United States, United Kingdom, European Union | - All other countries and regions |
| Study Design | - Original research, systematic reviews, meta-analyses - Toolkits, policies, guidelines, resources | - Conference abstracts, commentaries, letters |
| Year | - Published since 2011 | - Published prior to 2011 |
| Language | - English - French | - All other languages |

**Supplemental Table 2:** Peer-reviewed and grey literature documents by identification number, country and Canada’s Food Guide key message topics

| **ID** | **Reference** | **Country** | **General Healthy Eating** | **Vegetables and Fruit** | **Whole Grains** | **Protein Foods** | **Highly processed foods** | **Water** | **Sugary drinks** | **Marketing** | **Sponsorship** | **Cooking** | **Food culture/ traditions** | **Eat with Others** |
| --- | --- | --- | --- | --- | --- | --- | --- | --- | --- | --- | --- | --- | --- | --- |
| 1 | Boelsen-Robinson, T., Jerebine, A., Kurzeme, A., Gilham, B., Huse, O.T., Blake, M.R., Backholer, K., Chung, A., Peeters, A. (2021) Evaluating the implementation and customer acceptability of a sugar-sweetened beverage reduction initiative in thirty Australian aquatic and recreation centres. Public Health Nutrition, 24, 5166–75. | Australia |  |  |  |  |  |  | X |  |  |  |  |  |
| 2 | Clinton-McHarg, T., Gonzalez, S., Milner, S. et al. (2019) Implementing health policies in Australian junior sports clubs: an RCT.*BMC Public Health*19, 556. https://doi.org/10.1186/s12889-019-6873-3 | Australia | X |  |  |  |  | X |  |  |  |  |  |  |
| 3 | Hodges, B.C. (2017) Health Promotion at the ballpark: Peanuts, popcorn, and mighty molar. *Health Promotion Practice,* 18, 229–37. https://doi.org/10.1177/1524839916663684. | United States | X |  |  |  |  |  | X |  |  |  |  |  |
| 4 | ACT Government. (2020) Healthy food and drink choices. https://www.health.act.gov.au/about-our-health-system/population-health/health-promotion-programs/healthy-food-and-drink-choices (last accessed October 17 2022). | Australia | X |  |  |  |  | X |  | X | X |  |  |  |
| 5 | Active Monash.(2021) Sports club framework 2022-202. https://www.monash.vic.gov.au/files/assets/public/leisure/recreation-strategies/active-monash-sports-club-framework-2022-2027.pdf (last accessed November 14 2022) | Australia | X |  |  |  | X |  | X | X |  |  |  |  |
| 6 | Active Monash. (2020) Sports Club Framework 2021 - 2026. https://www.monash.vic.gov.au/files/assets/public/about-us/council/council-meetings/2020-26-may/2.3-attachment-council-meeting-26-may-2020.pdf (last accessed November 14 2022). | Australia |  |  |  |  |  |  |  | X |  |  |  |  |
| 7 | Stay Active Eat Healthy. (2021) Steps for offering healthy choices. https://stayactiveeathealthy.ca/managers/steps_for_offering_healthy_choices/  (last accessed November 14 2022). | Canada | X | X | X | X | X |  |  |  |  | X |  |  |
| 8 | Agencies for Nutrition Action Nga Takawaenga Hapai Kai. (2014) Healthy communities, healthy lives: New Zealand public health nutrition and physical activity sector vision 2024. https://ana.org.nz/wp-content/uploads/2016/08/Healthy-Communities-Healthy-Lives-FINAL.pdf (last accessed November 14 2022). | New Zealand |  |  |  |  |  |  |  | X |  |  |  |  |
| 9 | Agencies for Nutrition Action Nga Takawaenga Hapai Kai. (2016) Promoting healthy eating at the local government level. http://www.healthyaucklandtogether.org.nz/assets/Uploads/Resources/Promoting-Healthy-Eating-at-the-Local-Government-Level.pdf (last accessed November 14 2022). | New Zealand |  |  |  |  |  | X |  |  |  |  |  | X |
| 10 | Alberta Government. (2012) Alberta nutrition guidelines for children and youth: A childcare, school, and recreation/community centre resource manual. https://open.alberta.ca/dataset/1c291796-4eb0-4073-be8e-bce2d331f9ce/resource/3319786c-1df1-43ca-8693-067f733682dc/download/nutrition-guidelines-ab-children-youth.pdf (last accessed November 14 2022). | Canada | X | X | X | X | X | X |  | X |  |  |  |  |
| 11 | Alberta Health Services. (2015) Healthy vending toolkit. https://www.albertahealthservices.ca/assets/info/nutrition/if-nfs-healthy-vending-toolkit.pdf (last accessed November 14 2022). | Canada | X |  |  |  |  |  | X | X |  |  |  |  |
| 12 | Carrad, A. (2019) Developing health promoting sports clubs through organisational change. University of Wollongong, 2019. https://ro.uow.edu.au/cgi/viewcontent.cgi?article=1723&context=theses1 (last accessed November 14 2022) | Australia | X | X |  |  |  | X |  | X |  |  | X |  |
| 13 | Analysis and Policy Observatory.(2021) A toolkit for creating healthy food and drink. https://apo.org.au/node/311662 (last accessed November 14 2022). | Australia | X |  |  |  |  |  |  | X |  |  |  |  |
| 14 | Food-EPI Australia Project. Australian Federal government: summary of current government policy action to 8 May 2016. Melbourne: Deakin University, 2017. | Australia | X |  |  |  |  | X |  |  | X |  |  |  |
| 15 | Australian Federal Government. (2016) Healthy food environment policy index (Food-EPI) | Australia |  |  |  |  |  |  |  |  | X |  |  |  |
| 16 | Australian Capital Territory Government. (2017) Policies for tackling obesity and creating healthier food environments [PDF]. https://6caf7727-1efc-419a-8b13-d58f79d7351f.filesusr.com/ugd/2e3337_b0dd5841a9b143b7a032539919895e4b.pdf (last accessed November 14 2022). | Australia |  |  |  |  |  |  |  | X | X |  |  |  |
| 17 | Australia's Food Environment Dashboard. (2020) Sport and recreation. https://foodenvironmentdashboard.com.au/food-in-settings/sport-and-recreation/ (last accessed November 2022). | Australia |  |  |  |  |  |  | X | X |  |  |  |  |
| 18 | Basketball Victoria. (2016) Becoming a healthy choices association. https://basketballvictoria.com.au/wp-content/uploads/2016/03/FINAL-Becoming-a-Healthy-Eating-Association.pdf (last accessed November 2022). | Australia | X |  |  |  |  | X |  |  |  |  |  |  |
| 19 | BC Recreation & Parks Association.(2013) Getting Started Toolkit. https://www.bcrpa.bc.ca/media/154220/healthy-choices-toolkit_dec11-2014_web.pdf (last accessed November 2022). | Canada |  | X | X | X | X |  | X | X |  | X |  |  |
| 20 | Huse, O., Orellana, L., Ferguson, M., Palermo, C., Jerebine, A., Zorbas, C. (2012) Retailer-led healthy pricing interventions: a pilot study within aquatic and recreation centres in Victoria, Australia. *Health Promotion International,* 36, 430–48. | Australia |  |  |  |  |  |  |  | X |  |  |  |  |
| 21 | Kelly, B., Baur, L.A., Bauman, A.E., King, L., Chapman, K., Smith, B.J. (2011) Promoting health and nutrition through sport: Attitudes of the junior sporting community. Prevention Research Collaboration and Cancer Council, 2 March. https://ses.library.usyd.edu.au/bitstream/handle/2123/9072/PANORG_Kelly_Attitudes%20of%20the%20junior%20sporting%20community.pdf;jsessionid=54AF653DA95F862F8B4A2E004B2F71E2?sequence=1 (last accessed November 14 2022). | Australia | X | X |  |  |  |  |  |  | X |  |  |  |
| 22 | Kelly, B., King, L., Bauman, A.E., Baur, L.A., Macniven, R., Chapman, K., (2014) Identifying important and feasible policies and actions for health at community sports clubs: A consensus-generating approach. *Journal of Science and Medicine in Sport,* 17, 61–6. | Australia | X |  |  |  |  | X |  | X | X |  |  |  |
| 23 | Ontario Public Health Association. (2016) Getting started with healthy eating in your recreation setting. https://www.publichealthgreybruce.on.ca/Portals/0/Topics/Eating%20Well/NRC_OSNPPH_HE-rec-setting.pdf (last accessed November 14 2022). | Canada | X | X |  |  | X | X | X | X | X | X | X |  |
| 24 | Oxford County Public Health. (2016) A healthier recreation concession pilot project evaluation. http://www.oxfordcounty.ca/Portals/15/Documents/BB%20A%20Healthier%20Recreation%20Concession%20Pilot%20Evaluation%20July%202016.pdf (last accessed November 14 2022). | Canada |  |  |  |  |  |  |  | X |  |  |  |  |
| 25 | Power Up for Health. (2012) 'Mooving' towards healthier concessions in city of Edmonton recreation centers. https://abpolicycoalitionforprevention.ca/wp-content/uploads/2016/12/moos-mooving-toward-healthier-concessions-in-city-of-edmonton-recreation-centres-.pdf (last accessed November 14 2022). | Canada | X |  |  |  |  |  |  |  |  |  |  |  |
| 26 | Physical Activity, Nutrition and Obesity Research Group. (2016) Healthy food environment scoping review. https://ses.library.usyd.edu.au/bitstream/handle/2123/17007/155001%20Healthy%20Food%20Environment%20Report_FINAL.pdf?isAllowed=y&sequence=1 (last accessed November 14 2022). | Australia |  |  |  |  |  |  |  |  | X |  |  |  |
| 27 | Sacks, G., & Robinson, E. for the Food EPI Australia project team..(2019) Policies for tackling obesity and creating healthier food environments 2019 progress update Western Australian Government. https://preventioncentre.org.au/wp-content/uploads/2021/10/Food-Policy-Index-Progress-Update-2019-WA-FINAL-002.pdf (last accessed November 14 2022). | Australia |  |  |  |  |  |  |  | X | X |  |  |  |
| 28 | Western Australian Government. Healthy Food Environment Policy Index (Food-EPI) Australia – Progress update 2019 Western Australian Government. 2019 https://6caf7727-1efc-419a-8b13-d58f79d7351f.filesusr.com/ugd/2e3337_411f7f5361094e6f8e29dd64552b0872.pdf | Australia |  |  |  |  |  |  |  | X |  |  |  |  |
| 29 | Western Australian Government. (2017) Policies for tackling obesity and creating healthier food environments scorecard and priority recommendations for the Western Australian government. https://6caf7727-1efc-419a-8b13-d58f79d7351f.filesusr.com/ugd/2e3337_c36629fd837c4a908d9091bb69a65b78.pdf (last accessed November 14 2022). | Australia |  |  |  |  |  |  |  | X |  |  |  |  |
| 30 | Western Australian Government. Healthy Food Environment Policy Index (Food-EPI) – Australia 2016 Western Australian Government. 2016 | Australia | X |  |  |  |  | X |  | X |  |  |  |  |
| 31 | Wyndham City. (2020) Wyndham's sports facility user guide. https://www.wyndham.vic.gov.au/sites/default/files/2020-03/Sports%20Facility%20User%20Guide.pdf (last accessed November 14 2022). | Australia |  |  |  |  |  |  |  |  | X |  |  |  |
| 32 | Province of British Columbia. (2014) Healthier choices in vending machines in BC public buildings policy. https://stayactiveeathealthy.ca/about/ (last accessed November 14 2022). | Canada | X |  |  |  |  |  |  |  |  |  |  |  |
| 33 | Public Heath Law Center. (2015) From small steps to big leaps: promoting healthy food & beverage choices in parks & recreation facilities. https://publichealthlawcenter.org/sites/default/files/resources/fs-healthy-food-bev-parks-rec-WEB.pdf.pdf (last accessed November 14 2022). | United States |  | X | X | X | X | X | X |  |  |  |  |  |
| 34 | Public Health Nutrionists of Saskatchewan. Support Healthy Eating at Work and Play. 2010 http://www.fhhr.ca/Documents/SupportHealthyEating-July2010final.pdf | Canada | X |  |  |  |  |  |  | X |  |  |  |  |
| 35 | Queensland Government. (2019) Healthy food environment policy index (Food-EPI) Australia – Progress update 2019 Queensland Government. https://6caf7727-1efc-419a-8b13-d58f79d7351f.filesusr.com/ugd/2e3337_1e630b29d5924978b928865d0c6d50fd.pdf (last accessed November 14 2022). | Australia |  |  |  |  |  |  |  | X |  |  |  |  |
| 36 | Queensland Government.(2017) Policies for tackling obesity and creating healthier food environments scorecard and priority recommendations for the Queensland government. https://6caf7727-1efc-419a-8b13-d58f79d7351f.filesusr.com/ugd/2e3337_0f661f0fd279438297c96e3fc61a1a53.pdf (last accesssed November 14 2022). | Australia |  |  |  |  |  |  |  | X |  |  |  |  |
| 37 | Queensland Government. (2016) Healthy food environment policy index (Food-EPI) – Australia 2016 Queensland Government. | Australia |  |  |  |  |  |  |  | X | X |  |  |  |
| 38 | Queensland Government. (2021) About the food for sport guidelines. https://www.qld.gov.au/recreation/sports/club-support/food-for-sport/about-the-food-for-sport-guidelines (last accessed November 13 2022). | Australia | X |  |  |  | X |  |  |  |  |  |  |  |
| 39 | Recreation PEI. (2020) Healthy eating in PEI rereation setings: Action starter kit. https://recreationpei.ca/healthy-eating-in-recreation-facilities/ (last accessed November 13 2022). | Canada |  |  |  |  |  | X | X | X |  |  |  |  |
| 40 | Regional District of Nanaimo. (2021) Healthy choices philosophy and guiding principles. https://www.rdn.bc.ca/healthy-choices-philosophy (last accessed November 13 2022). | Canada | X |  |  |  | X |  |  | X |  |  |  |  |
| 41 | Australian Federal Government. (2017) Policies for tackling obesity and creating healthier food environments scorecard and priority recommendations for the Australian Federal government. https://6caf7727-1efc-419a-8b13-d58f79d7351f.filesusr.com/ugd/2e3337_ef62a61c075f402ba80bcdd1df8174ae.pdf (last accessed November 14 2022). | Australia |  |  |  |  |  |  |  |  | X |  |  |  |
| 42 | Australian Capital Territory (ACT) Government. (2019) Policies for tackling obesity and creating healthier food environments 2019 progress update Australian Capital Territory (ACT) Government. https://6caf7727-1efc-419a-8b13-d58f79d7351f.filesusr.com/ugd/2e3337_859f2458b037469ca46127763404418b.pdf (last accessed November 14 2022). | Australia |  |  |  |  |  |  |  | X | X |  |  |  |
| 43 | South Australian Government. (2019) Policies for tackling obesity and creating healthier food environments: 2019 progress update, South Australian Government. South Australian Government.. https://preventioncentre.org.au/wp-content/uploads/2021/10/Food-Policy-Index-Progress-Update-2019-SA-FINAL-002.pdf (last accessed November 14 2022) | Australia | X |  |  |  |  |  |  | X | X |  |  |  |
| 44 | South Australian Government. (2016) Healthy food environment policy index (Food-EPI) – Australia 2016 South Australian Government. | Australia |  |  |  |  |  |  |  | X |  |  |  |  |
| 45 | South Metro Junior Football League. (2021) South metro junior football league (SMJFL) Healthy choices canteen's policy. https://smjfl.com.au/wp-content/uploads/2021/03/SMJFL-HC-Canteens-Policy.pdf (last accessed November 14 2022). | Australia | X |  |  |  | X |  |  | X |  |  |  |  |
| 46 | Southern Melbourne Primary Care Partnership. (2018) Healthy club canteens toolkit. http://smpcp.org.au/index.php/our-work/projects/healthy-eating/healthy-club-canteens-toolkit/152-smpcp/517-hcc-drinks (last accessed November 14 2022). | Australia | X |  |  |  |  |  | X | X |  |  |  |  |
| 47 | Sport Waikato. (2018) Food and nutrition policy. https://www.sportwaikato.org.nz/SportWaikato/media/SW/Document%20Resource/sportwaikato-foodandnutritionpolicy.pdf (last accessed November 14 2022). | New Zealand |  | X | X | X |  | X | X | X |  |  | X |  |
| 48 | SportMed BC. (2010) Guidelines for food and beverages available at sporting events in BC. https://www.bcsoccer.net/files/Coach/SoccerScience/Sporting_event_guidelines_FINAL_june2010.pdf (last accessed November 14 2022). | Canada | X |  |  |  | X |  | X |  |  |  |  |  |
| 49 | Surf Coast Shire. Wurdi Baierr Stadium Café Sub-licence. | Australia | X |  |  |  | X | X |  | X | X |  |  |  |
| 50 | Tasmanian Government. (2019) Policies for tackling obesity and creating healthier food environments 2019 progress update Tasmanian Government. https://6caf7727-1efc-419a-8b13-d58f79d7351f.filesusr.com/ugd/2e3337_000c1656f1ab43e09d355b92bf1231ac.pdf (last accessed November 14 2022). | Australia |  |  |  |  |  |  |  | X | X |  |  |  |
| 51 | Tasmanian Government. (2016) Healthy food environment policy index (Food-EPI) – Australia 2016 Tasmanian Government. | Australia |  |  |  |  |  |  |  | X |  |  |  |  |
| 52 | Town of Antigonish. (2016) Municipal food and nutrition policy - Town of Antiognish. https://www.townofantigonish.ca/policies-bylaws/policies/66-healthy-eating-policy/file.html (last accessed November 14 2022). | Canada | X |  |  |  |  |  | X | X | X |  |  |  |
| 53 | Town of Fort Smith. (2018) The corporation of town of Fort Smith policy and procedures manual policy: Healthy foods policy. https://fortsmith.ca/sites/default/files/CP%20314%20Healthy%20Foods%20Policy.pdf (last accessed November 14 2022). | Canada |  |  |  |  |  |  | X | X | X |  |  |  |
| 54 | Town of Harbour Grace. (2019) Municipal healthy eating policy. https://www.hrgrace.ca/parks-recreation/municipal-healthy-eating-policy/ (last accessed November 14 2022). | Canada |  | X | X | X | X | X | X |  |  |  |  |  |
| 55 | Town of Yarmouth. (No Date) Healthy Eating in municipal and recreation settings policy. https://www.google.com/url?sa=t&rct=j&q=&esrc=s&source=web&cd=&ved=2ahUKEwjYnLfKzbrzAhVYSvEDHYB3DOc4ChAWegQIDRAB&url=https%3A%2F%2Fwww.townofyarmouth.ca%2F578-healthy-eating-in-municipal-and-recreation-settings-policy-1%2Ffile.html&usg=AOvVaw1UExG8U7aIgcVQexwgURsX (last accessed November 14 2022). | Canada | X |  |  |  |  | X |  | X | X |  |  |  |
| 56 | University of Alberta. (2019) Lethbridge's recreation facilities 2018/2019 nutrition report card summary. https://agendas.lethbridge.ca/AgendaOnline/Documents/ViewDocument/ATTACHMENT%204%20-%20NUTRITION%20REPORT.pdf?meetingId=2640&documentType=Agenda&itemId=70814&publishId=57456&isSection=false (last accessed November 14 2022). | Canada | X |  |  |  | X | X |  |  |  |  |  |  |
| 57 | University of Missouri. (2015) Eat smart in parks toolkit. https://extension.missouri.edu/media/wysiwyg/Extensiondata/Pub/pdf/hesguide/foodnut/n02051.pdf (last accessed November 14 2022). | United States |  | X | X | X | X | X | X | X |  |  |  |  |
| 58 | University of Toronto. (2017) Healthy food envionment policy index (Food-EPI) Ontario. https://labbelab.utoronto.ca/wp-content/uploads/2017/12/Evidence-Document-ON-reformat-Nov-8.pdf (last ccessed November 14 2022). | Canada |  |  |  |  |  |  |  | X |  |  |  |  |
| 59 | Vandevijvere, S., Mackay, S., D’Souza, E., Swinburn, B. (2018) How healthy are New Zealand food environments? University of Auckland. https://figshare.com/s/f877a2b8b8129d456bb4 (last accessed November 14 2022) | New Zealand |  |  |  |  |  |  |  | X | X |  |  |  |
| 60 | VicHealth. (2018) Healthy food and drink choices in community sport: Building on success. https://www.vichealth.vic.gov.au/-/media/ResourceCentre/PublicationsandResources/healthy-eating/Healthy-Choice/Evaluation-summary-healthy-food-and-drink-choices-in-community-sport.pdf (last accessed November 14 2022). | Australia |  |  |  |  |  | X | X | X |  |  |  |  |
| 61 | VicHealth. (2014) Healthy sporting environments demonstration project. https://www.latrobe.edu.au/__data/assets/pdf_file/0011/741854/HSEDP_evaluation-highlights.pdf (last accessed November 14 2022). | Australia | X |  |  |  |  |  |  |  | X |  |  |  |
| 62 | VicHealth. (2016) City of Melbourne sports and recreation centre healthy food retail case study. https://www.vichealth.vic.gov.au/-/media/ResourceCentre/PublicationsandResources/healthy-eating/Healthy-Choice/City-of-Melbourne-sports-and-recreation-centre-healthy-food-retail-case-study.pdf (last accessed November 14 2022). | Australia |  |  |  |  | X |  |  |  |  |  |  |  |
| 63 | VicHealth. (2010) Evaluation report: Healthy club canteens project. https://www.vichealth.vic.gov.au/-/media/ProgramsandProjects/HealthyEating/Healthy-Club-Canteens-project/HealthyClubEvaluationReport_no_appendices.pdf?la=en&hash=0BDD622E0B324D1DCAC1538F912EC42A6B28AE7A (last accessed November 14 2022). | Australia | X |  |  |  | X |  |  | X |  |  |  |  |
| 64 | VicHealth. (2015) Strategies to influence healthy practices in community sports settings. https://goodcdn.app/memberhq/vicsport/uploads/Strategies-Document-Healthy-Choices---Final-with-cover.pdf (last accessed November 14 2022). | Australia | X |  |  |  |  | X | X | X | X |  |  |  |
| 65 | Vicsport. (2015) Strategies to influence healthy practices in community sport settings. https://www.sportsfocus.com.au/wp-content/uploads/2015/10/Hydration-Strategies1.pdf (last accessed November 14 2022). | Australia |  |  |  |  |  | X | X | X | X |  |  |  |
| 66 | Victoria State Government. (2016) Healthy choices: policy guidelines for sport and recreation centres. https://www.google.com/url?sa=t&rct=j&q=&esrc=s&source=web&cd=&ved=2ahUKEwib9-Dn_afzAhXqmeAKHcpiAjI4ChAWegQIEBAB&url=https%3A%2F%2Fwww2.health.vic.gov.au%2FApi%2Fdownloadmedia%2F%257B7EF29993-EEC4-414F-B942-ADF75729E734%257D&usg=AOvVaw3awAeTOQy_x-g3V4aCE2e3 (last accessed November 14 2022). | Australia | X |  |  |  | X | X |  | X | X |  |  |  |
| 67 | Victoria State Government. (2019) Healthy choices in sport and recreation facilities. https://sport.vic.gov.au/__data/assets/pdf_file/0038/56999/healthy20choices20in20sport20and20recreation20facilities20-20implementation20guidelines20and20template.pdf (last accessed November 14 2022). | Australia | X |  |  |  | X | X |  | X | X |  |  | X |
| 68 | Victorian Government.(2017) Healthy food environment policy index (Food-EPI) – Australia 2016. https://www.opc.org.au/downloads/food-policy-index/Food-EPI-Australia-2016-Victoria-policy-details.pdf (last accessed November 14 2022). | Australia |  |  |  |  |  |  |  | X |  |  |  |  |
| 69 | Victorian Government. (2019) Policies for tackling obesity and creating healthier food environments 2019 progress update Victorian Government. https://6caf7727-1efc-419a-8b13-d58f79d7351f.filesusr.com/ugd/2e3337_a03e54e09d7e444d9b69817b1ca4883a.pdf Victorian Government. Healthy Food Environment Policy Index (Food-EPI) Australia – Progress update 2019 Victorian Government. 2019 https://6caf7727-1efc-419a-8b13-d58f79d7351f.filesusr.com/ugd/2e3337_f805fff887114733a81ae9cacb0a7ce7.pdf (last accessed November 14 2022). | Australia |  |  |  |  |  |  |  | X | X |  |  |  |
| 70 | Victorian Government. (2016) Healthy food environment policy index (Food-EPI) – Australia 2016 Victorian Government. | Australia |  |  |  |  |  |  |  | X |  |  |  |  |
| 71 | Arrondissement de Côte-des-Neiges – Notre-Dame-de-Grâce. (2010). Politique en faveur des saines habitudes de vie. Volet 1 - Saine alimentation et élimination des gras trans dans les établissements municipaux relevant de l'arrondissement. https://web.archive.org/web/20220630143358/https://www.cqpp.qc.ca/documents/file/2012/pol_alim_cdn-ndg.pdf (last accessed January 18 2023) | Canada | X |  |  |  |  |  |  |  |  |  |  |  |
| 72 | Healthy Eating Physical Activity Coalition of New Brunswick. (2018) Des aliments sains dans les installations récréatives. https://nanopdf.com/download/des-aliments-sains-dans-les-installations-recreatives-le-simple-bon_pdf (last accessed November 14 2022). | Canada |  |  |  |  |  |  |  | X |  |  |  |  |
| 73 | Maski Quebec. (2016) Ensemble pour manger mieux. https://www.maski.quebec/wp-content/uploads/2018/02/cadre-reference-saine-alimentation-mrc-maskinonge.pdf (last accessed November 14 2022). | Canada |  |  |  |  |  |  |  |  |  |  |  |  |
| 74 | Ville de Gatineau. (2012) Gatineau's nutrition policy for healthy choices! https://www.gatineau.ca/docs/guichet_municipal/administration_municipale/politiques_vision/politique_alimentaire.en-CA.pdf (last accessed November 14 2022). | Canada |  | X |  |  | X | X | X |  |  |  |  |  |
| 75 | Ville de Rimouski. (2016) Saveur plaisir equilibre. https://rimouski.ca/storage/app/media/ville/decouvrir/publications-et-plan-daction/politiques-generales/Politique_alimentaire.pdf (last accessed November 14 2022). | Canada | X |  |  |  |  |  |  |  |  |  |  |  |
| 76 | Campaspe Primary Care Partnership. (2020) Sporting club toolkit. https://campaspepcp.com.au/wp-content/uploads/2021/02/Sporting-Club-Toolkit_ERH-2020.pdf (last accessed November 14 2022). | Australia | X |  |  |  |  | X | X | X | X |  |  |  |
| 77 | Christchurch City Council. (2017) Healthy food action plan 2017. https://foodsystemsplanning.ap.buffalo.edu/wp-content/uploads/sites/68/2019/05/Healthy-Food-Action-Plan-2017-05-11.pdf (last accessed November 14 2022). | New Zealand | X |  |  |  |  | X |  |  | X |  |  |  |
| 78 | City of Darwin. (2019) Recreation and healthy lifestyle. https://www.darwin.nt.gov.au/sites/default/files/publications/attachments/policy_no_046_-_recreation_and_healthy_living_1.pdf (last accessed November 14 2022). | Australia | X |  |  |  |  |  |  |  |  |  |  |  |
| 79 | City of Greater Bendigo. (2020) City of Greater Bendigo healthy facilities policy. https://www.bendigo.vic.gov.au/sites/default/files/2020-10/Draft%20Healthy%20Facilities%20Policy.pdf (last accessed November 14 2022). | Australia | X |  |  |  |  | X |  | X | X |  |  |  |
| 80 | City of Missisauga. (2020) Healthy food and beverage policy for recreation facilities. https://www7.mississauga.ca/documents/committees/general/2020/2020_03_11_General_Committee_Agenda.pdf#page=5 (last accessed November 14 2022). | Canada |  | X | X | X | X | X |  |  |  | X |  |  |
| 81 | City of Prince George. (2011) Healthy food and beverage sales - Recreation facilities. https://princegeorge.ca/City%20Hall/Documents/Mayor%20and%20Council/Council_Procedure_Healthy_Food_and_Beverage_Sales_Recreation_Facilities.pdf (last accessed November 14 2022). | Canada |  |  |  |  |  |  |  |  |  |  |  |  |
| 82 | Cockburn Basketball Association. (2021) Healthy sporting club policy. https://cougarfamily.com/wp-content/uploads/2021/01/CBA-Healthy-Sporting-Clubs-Policy.pdf (last accessed November 14 2022). | Australia | X |  |  |  |  | X |  | X |  |  |  |  |
| 83 | Eat Great and Participate. (No date) Making the move to healthy choices. https://stayactiveeathealthy.ca/wp-content/uploads/formidable/6/Making-the-Move-to-Healthy-Choices-A-Healthy-Eating-Toolkit-for-Recreation-Sport-and-Community-Food-Service-Providers.pdf (last accessed November 14 2022). | Canada | X | X | X | X | X | X |  | X |  |  |  |  |
| 84 | Healthy Eating Physical Activity Coalition of New Brunswick. (2016) Healthy foods in recreation facilities. https://nutritionconnections.ca/wp-content/uploads/2021/02/nanopdf.com_healthy-foods-in-recreation-facilities.pdf (last accessed November 14 2022). | Canada |  |  |  |  |  |  |  | X |  |  |  |  |
| 85 | Government of South Australia. Creating Healthier Local Food Environments [PDF]. No date. Available from: https://www.sahealth.sa.gov.au/wps/wcm/connect/01e6ae8042515e6788598eadc5e910c3/17021.5+Creating+Health+Local+Food+Enviro+ONLINE_FINAL.pdf?MOD=AJPERES&amp;CACHEID=ROOTWORKSPACE-01e6ae8042515e6788598eadc5e910c3-nKL-TAL | Australia |  |  |  |  |  | X |  |  |  |  |  |  |
| 86 | Harvard T.H. Chan School of Public Health. Healthy Food Environment Recommendation: Complete list [PDF]. 2021. Available from: https://www.hsph.harvard.edu/obesity-prevention-source/obesity-prevention/food-environment/healthy-food-environment-recommendations-for-obesity-prevention-complete-list/ | United States | X |  |  |  |  | X | X | X |  |  |  |  |
| 87 | Healthway. (2018) Healthy venues project. https://www.healthway.wa.gov.au/wp-content/uploads/Healthy-Venues-Project-Guidelines-FINAL.pdf (last accessed November 14 2022). | Australia | X |  |  |  |  |  |  |  |  |  |  |  |
| 88 | Healthy Auckland Together. (2017) The healthy Auckland together plan 2015-2020. http://www.healthyaucklandtogether.org.nz/assets/Uploads/HAT-actionplan.pdf (last accessed November 14 2022). | New Zealand |  |  |  |  |  |  |  |  |  |  |  |  |
| 89 | Parks Victoria. (2016) Healthy choices: Policy guidelines for parks. https://www.health.vic.gov.au/preventive-health/healthy-choices (last accessed November 14 2022). | Australia | X |  |  |  | X | X |  | X | X | X |  |  |
| 90 | Healthy Families BC. (2013) Guidelines for food & beverage sales in BC. http://healthyeatingatschool.ca/uploads/2013_food_guidelines_updated.pdf (last accessed November 14 2022). | Canada | X |  |  |  | X | X |  | X |  |  | X |  |
| 91 | Katunga Football-Netball Club. (2021) Food and nutrition policy. https://websites.mygameday.app/get_file.cgi?id=36769438 (last accessed November 14 2022). | Australia | X |  |  |  |  | X |  | X | X | X |  |  |
| 92 | Keilor Park Sports Club. (2019) Healthy food and drink policy. https://www.keilorparksportsclub.com.au/wp-content/uploads/2019/09/KPSC-Healthy-Food-Drink-Policy.pdf (last accessed November 14 2022). | Australia | X |  |  |  | X | X |  | X | X |  |  |  |
| 93 | Leeds, Grenville & Lanark Health District. (2017) Fuelling recreation in your community toolkit. https://healthunit.org/wp-content/uploads/Fuelling_Recreation_in_Your_Community.pdf (last accessed November 14 2022). | Canada | X |  |  |  |  |  | X | X |  | X |  |  |
| 94 | Michigan Recreation & Parks Association. (2015) Eat well mParks toolkit. https://cdn.ymaws.com/www.mparks.org/resource/resmgr/programs_&_initiatives/health_&_wellness/eatwell_mparks_toolkit.pdf (last accessed November 14 2022). | United States | X |  |  |  | X | X | X | X |  |  |  |  |
| 95 | Minneapolis Parks and Recreation Board. (2021) Healthy food policy. https://www.minneapolisparks.org/activities__events/healthy_food_policy/ (last accessed November 14 2022). | United States | X |  |  |  |  |  |  |  |  |  |  |  |
| 96 | Move to Healthy Choices Committee. (2012) Nutrition policy for community recreation facilities. https://stayactiveeathealthy.ca/wp-content/uploads/formidable/6/Nutrition-Policy-Guidelines-Aug-2017MB.pdf (last accessed November 14 2022). | Canada | X | X | X |  | X |  | X | X |  | X |  |  |
| 97 | National Alliance for Nutrition and Activity. (2020) National alliance for nutrition and activity model nutrition standards for “Grab-and-Go” foods and beverages. https://cspinet.org/sites/default/files/NANA_Model_Nutrition_Standards_Grab_and_Go_6-15-20_0.pdf (last accessed November 14 2022). | United States | X |  |  |  |  | X | X |  |  |  |  |  |
| 98 | New South Wales (NSW) Government. (2019) Policies for tackling obesity and creating healthier food environments 2019 progress update New South Wales (NSW) Government. https://6caf7727-1efc-419a-8b13-d58f79d7351f.filesusr.com/ugd/2e3337_ddaaefc9382f489fb03a5e19f9c4ef90.pdf (last accessed November 14 2022). | Australia |  |  |  |  |  |  |  |  | X |  |  |  |
| 99 | New South Wales (NSW) Government. (2017) Policies for tackling obesity and creating healthier food environments scorecard and priority recommendations for the New South Wales government. https://6caf7727-1efc-419a-8b13-d58f79d7351f.filesusr.com/ugd/2e3337_ce14f604670a41f48bd59ea898f2ff5c.pdf (last accessed November 14 2022). | Australia |  |  |  |  |  |  |  |  | X |  |  |  |
| 100 | North Carolina Recreation and Park Association. (No date) Healthy snack guidelines. https://www.ncrpa.net/page/SnackGuidelines/Healthy-Snack-Guidelines-and-Policy.htm (last accessed November 14 2022). | United States | X |  | X | X |  |  |  | X |  | X |  |  |
| 101 | Northern Territory Government. (2017) Healthy food environment policy index (Food-EPI) – Australia 2016. https://www.opc.org.au/downloads/food-policy-index/Food-EPI-Australia-2016-Northern-Territory-policy-details.pdf (last accessed November 14 2022). | Australia | X |  |  |  |  |  |  | X | X |  |  |  |
| 102 | Northern Territory Government. (2019) Policies for tackling obesity and creating healthier food environments 2019 progress update Northern Territory Government. https://6caf7727-1efc-419a-8b13-d58f79d7351f.filesusr.com/ugd/2e3337_9fc67775a7da4f338a4ed566a35c3ee3.pdf (last accessed November 14 2022). | Australia |  |  |  |  |  |  |  | X | X |  |  |  |
| 103 | Nova Scotia Department of Health and Wellness. (2014) Healthy eating in recreation and sports settings guidelines. http://www.rfans.com/wp-content/uploads/2016/08/FINAL-HERSS-Guidelines-v0-3.pdf (last accessed November 14 2022). | Canada | X |  |  |  | X | X | X | X | X | X | X |  |
| 104 | Ruggiero, C.F., Poirier, L., Trude, A.C.B., Yang, T., Schwendler, T., Gunen. B., Loh, I.H., Perepezko, K., Nam, C.S., Sato, P., Gittelsohn. J. (2018) Implementation of B’more healthy communities for kids: Process evaluation of a multi-level, multi-component obesity prevention intervention. *Health Education Research,* 33, 458-472. | United States | X |  | X |  | X | X | X | X |  |  |  |  |
| 105 | Silberfarb, L.O., Savre, S., Geber, G. (2014) An approach to assessing multicity implementation of healthful food access policy, systems, and environmental changes. *Preventing Chronic Disease*, 11, E64. | United States | X |  |  |  |  | X | X |  |  |  |  |  |
| 106 | Northern Territory Government. (2016) Healthy food environment policy index (Food-EPI) – Australia 2016 Northern Territory government. | Australia | X |  |  |  |  |  |  | X | X |  |  |  |
| 107 | New South Wales Government. (2016) Healthy food environment policy index (Food-EPI) – Australia 2016 New South Wales government. | Australia |  |  |  |  |  |  |  | X | X |  |  |  |
| 108 | International Network for Food and Obesity/NCDs Research, Monitoring and Action Support (INFORMA) & University of Auckland (2019). INFORMAS. (2019). Benchmarking New Zealand food environment policies against international best practice: Evidence summary for expert panel Food-EPI 2017-2019.. | New Zealand |  |  |  |  |  |  |  | X | X |  |  |  |
| 109 | Boelsen-Robinson, T., Jerebine, A., Kurzeme, A., Gilham. B., Huse. O.T., Blake, M.R., Backholer, K., Chung, A., Peeters, A. (2021) Evaluating the implementation and customer acceptability of a sugar-sweetened beverage reduction initiative in thirty Australian aquatic and recreation centres. *Public Health Nutrition,* 24, 5166–75. | Australia |  |  |  |  |  |  | X |  |  |  |  |  |
| 110 | Warken, M., Sanden, T., Shanks, N., Engler-Stringer, R., & Vatanparast, H. (2022). A need for multi-sector and multi-pronged solutions to address the many barriers inhibiting change from unhealthy food environments in publicly funded recreation facilities: a mixed-method study. *Applied Physiology, Nutrition, and Metabolism / Physiologie Appliquee, Nutrition et Metabolisme*, 47(8), 847–859. | Canada | X |  |  |  |  |  |  | X |  |  |  |  |
| 111 | Blake MR, Romaniuk H, Riesenberg D, et al. (2022). Implementation and sales impact of a capacity building intervention in Australian sporting facility food outlets: a longitudinal observational study. *BMJ Nutrition, Prevention & Health,* 5. | Australia |  |  |  |  |  | X | X |  |  |  |  |  |
| 112 | Blake, M.R., Boelsen-Robinson, T., Hanna, L., Ryan, A., Peeters, A. (2021) Implementing a healthy food retail policy: a mixed-methods investigation of change in stakeholders’ perspectives over time. *Public Health Nutrition*, 24, 2669-80. | Australia | X |  |  |  |  |  |  |  |  |  |  |  |
| 113 | Boelsen-Robinson T, Orellana L, Backholer K, Kurzeme A, Jerebine A, Gilham B, Chung A, Peeters A. Change in drink purchases in 16 Australian recreation centres following a sugar-sweetened beverage reduction initiative: an observational study. BMJ open. 2020 Mar 1;10(3):e029492. | Australia |  |  |  |  |  |  | X | X |  |  |  |  |
| 114 | Breeze, P., Womack, R., Pryce, R., Brennan, A., Goyder, E. (2018) The impact of a local sugar sweetened beverage health promotion and price increase on sales in public leisure centre facilities. *PLoS One,* 13, e0194637. | United Kingdom |  |  |  |  |  | X | X |  |  |  |  |  |
| 115 | Economos, C.D., Anzman-Frasca, S., Koomas, A.H., Chan, G., Folta, S.C., Heck, J., Newman, M., Sacheck, J.M. (2017) Snacks, beverages, and physical activity during volunteer-led out-of-school-time programs: a cross-sectional analysis. *BMC Public Health*, 17, 1-0. | United States |  | X |  |  | X | X | X |  |  |  |  |  |
| 116 | Gonzalez S, Clinton‐McHarg T, Kingsland M, Hall A, Lecathelinais C, Milner S, Sherker S, Rogers B, Doran C, Brooke D, Wiggers J. Promotion of healthy eating in clubs with junior teams in Australia: A cross‐sectional study of club representatives and parents. Health Promotion Journal of Australia. 2019 Dec;30:15-9. | Australia |  | X |  |  |  | X |  | X | X |  |  |  |
| 117 | Lawman, H.G., Lofton, X., Grossman, S., Root, M., Perez, M., Tasian, G. (2019) A randomized trial of a multi-level intervention to increase water access and appeal in community recreation centers. *Contemporary Clinical Trials,* 79, 14–20. | United States |  |  |  |  |  | X |  | X |  |  |  |  |
| 118 | Lloyd, B., Dumbrell, S. (2011) Modifying the food supply at a community swimming pool: a case study. *Health Promotion Journal of Australia,* 22, 22–6. | Australia | X |  |  |  |  |  |  | X |  |  |  |  |
| 119 | McFadyen, T., Chai, L.K., Wyse, R., Kingsland, M., Yoong, S.L., Clinton-McHarg. T. (2018) Strategies to improve the implementation of policies, practices or programmes in sporting organisations targeting poor diet, physical inactivity, obesity, risky alcohol use or tobacco use: a systematic review. *BMJ Open,* 8, e019151. | Australia |  | X |  |  |  |  | X | X |  |  |  |  |
| 120 | D McIsaac, J.L., Jarvis, S., Lee Olstad, D., Naylor, P., Rehman, L., FL Kirk, S. (2018) Voluntary nutrition guidelines to support healthy eating in recreation and sports settings are ineffective: findings from a prospective study. *AIMS Public Health,* 5, 411–20. | Canada | X | X | X |  | X | X |  |  |  |  |  |  |
| 121 | Milner, S., Sherker, S., Clinton-McHarg, T., Dray, J., Zukowski, N., Gonzalez, S., Kingsland, M., Ooi, J. Y., Murphy, A., Brooke, D., Wiggers, J., & Wolfenden, L. (2018) Cluster randomised controlled trial of a multicomponent intervention to support the implementation of policies and practices that promote healthier environments at junior sports clubs: study protocol. *BMJ open*, 8, e018906. | Australia | X |  |  |  |  |  |  | X |  |  |  |  |
| 122 | Naylor, P-J., Wekken, S.V., Trill, D., Kirbyson, A. (2015) Facilitating healthier food environments in public recreation facilities: Results of a pilot roject in British Columbia, Canada. *Journal of Park and Recreation Administration,* 28. | Canada | X |  |  |  | X |  |  | X |  |  |  | X |
| 123 | Wolfenden, L., Kingsland, M., Rowland, B.C, Dodds, P., Gillham, K., Yoong. S.L. (2015) Improving availability, promotion and purchase of fruit and vegetable and non sugar-sweetened drink products at community sporting clubs: a randomised trial. *International Journal of Behavavioural Nutrition and Physical Activity,* 12, 35. | Australia |  | X |  |  |  |  | X | X |  |  |  |  |
| 124 | Wright-Pedersen, S., Chalton, K., Kelly, B., Whalan, M. (2017) Pilot testing a healthy canteen initiative in the junior football club setting using the health star rating approach. *Nutrition Diet,* 17, 50–86. | Australia | X |  |  |  |  |  |  | X |  |  |  |  |
| 125 | Lane, C., Naylor, P.J., Tomlin, D., Kirk, S., Hanning, R., Masse, L., Olstad, D.L., Prowse, R., Caswell, S., Jarvis, S., Milford, T. (2019) Healthy vending contracts: Do localized policy approaches improve the nutrition environment in publicly funded recreation and sport facilities?. *Preventive Medicine Reports,* 16, 100967 | Canada |  |  |  |  |  |  |  |  |  |  |  |  |
| 126 | Olstad, D.L., Poirier, K., Naylor, P.J., Shearer, C., Kirk, S.F. (2015) Policy outcomes of applying different nutrient profiling systems in recreational sports settings: the case for national harmonization in Canada. *Public health Nutrition,* 18, 2251-62. | Canada | X |  |  |  |  |  |  |  |  |  |  |  |
| 127 | Olstad, D.L, Prowse, R.J., Raine, K.D., Tomlin, D., Kirk, S.F., McIsaac, J.L., Mâsse, L.C., Caswell, M.S., Hanning, R.M., Milford, T., Naylor, P.J. (2020) Baseline results from the Eat, Play, Live trial: A randomized controlled trial within a natural experiment examining the role of nutrition policy and capacity building in improving food environments in recreation and sport facilities. *Food Policy,* 92, 101870. | Canada | X | X |  |  | X |  |  |  |  | X |  |  |
| 128 | Olstad, D.L., Goonewardene, L.A., McCargar, L.J., Raine, K.D. (2014) Choosing healthier foods in recreational sports settings: a mixed methods investigation of the impact of nudging and an economic incentive. *International Journal of Behavioral Nutrition and Physical Activity,* 11, 1-4. | Canada |  |  |  |  |  |  |  | X |  |  |  |  |
| 129 | Olstad, D.L., Vermeer, J., McCargar, L.J., Prowse, R.J., Raine, K.D. (2015) Using traffic light labels to improve food selection in recreation and sport facility eating environments. *Appetite,* 91, 329-35. | Canada |  |  |  |  |  |  |  | X |  |  |  |  |
| 130 | Prowse, R.J., Naylor, P.J., Olstad, D.L., Carson, V., Mâsse, L.C., Storey, K., Kirk, S.F., Raine, K.D. (2018) Reliability and validity of a novel tool to comprehensively assess food and beverage marketing in recreational sport settings. *International Journal of Behavioral Nutrition and Physical Activity,* 15, 1-2. | Canada | X |  |  |  |  |  |  | X |  |  |  |  |
| 131 | Prowse, R.J., Tomlin, D.L., Naylor, P.J., Raine, K.D. (2018) Exploring nutrition labelling of food and beverages in vending machines in Canadian recreational sport settings. *Canadian Journal of Dietetic Practice and Researc,* 80, 55-62. | Canada |  |  |  |  |  |  |  | X |  |  |  |  |
| 132 | Van Rookhuijzen M., & De Vet E.Nudging healthy eating in Dutch sports canteens: A multi-method case study. Public Health Nutr. 2020 September;24(2): 327-337. | Netherlands |  |  |  |  |  |  | X | X |  |  |  |  |

**Supplemental Table 3: Document type by Canada’s Food Guide key message topics**

| **Document Type** | General Healthy Eating | Vegetables and Fruit | Whole Grains | Protein Foods | Highly processed foods | Water | Sugary drinks | Marketing | Sponsorship | Cooking | Food culture/traditions | Eat with others |
| --- | --- | --- | --- | --- | --- | --- | --- | --- | --- | --- | --- | --- |
| Toolkits (n=11) | 9 | 5 | 4 | 3 | 7 | 6 | 9 | 11 | 3 | 4 | 1 | 1 |
| Practices (n=33) | 15 | 5 | 2 | 0 | 6 | 8 | 10 | 21 | 1 | 0 | 1 | 1 |
| Policies (n=37) | 25 | 7 | 4 | 4 | 11 | 20 | 10 | 19 | 17 | 4 | 1 | 0 |
| Guidelines and recommendations (n=51) | 19 | 3 | 3 | 3 | 8 | 13 | 8 | 36 | 20 | 3 | 2 | 1 |
| Total (n=132) | 68 | 20 | 13 | 10 | 32 | 46 | 37 | 87 | 41 | 11 | 5 | 3 |
|  | 51.5% | 15.2% | 9.8% | 7.6% | 24.4% | 34.8% | 28.0% | 65.9% | 31.1% | 8.3% | 3.8% | 2.3% |

**Supplemental Table 4: Indicators for Food and Beverage Marketing in Recreation and Sport Settings**  according­ to the HEERSS framework

| HEERSS  Component | Indicators for Marketing |
| --- | --- |
| Profile | - Healthy food is promoted (4,10,13,14,18,19,20,22,49,52,55,64,66, 67,82,83,84,87,89,92,94,96,100,101, 104,117,123,128,132) - Unhealthy foods and beverages promotion is restricted (4,13,14, 27,28,29,30,35,36,37,42,43,44,49,59, 60,64,65,66,67,79,82,87, 89,101,103,133) |
| Portion | N/A |
| Pricing | See Priming – Rewards and Incentives |
| Promotion | Food marketing   - Restrict unhealthy food and beverage marketing in sport or children’s settings (16,23,27,28,29,30,35,36,37,42,43,44,50,51,58,68,69,70,87, 98,99,101,102,103,106,107,108) - Restrict unhealthy food and beverage marketing to children and youth (39,44,49,51,56,66,67,70,89,101,106,107,108) - Restrict any food marketing to children and youth (23,39) - Limit marketing to children to healthy foods (19) - Restricting all promotion, including sponsorship, related to unhealthy food and beverages is a condition to receive funding (16,27,42,43,50,69,98,99,102)   sponsorship   - Restrict unhealthy food and beverage sponsorship (16,21,22,26,27,31, 37,41,42,42,49,50,53,66,67,69,89,98,99,102,106,107,108,116)   - E.g. Fast Food sponsors (21, 116)   - E.g. Sugary drink, confectionery, supermarket sponsors (21) - Restrict any food-related sponsorship of children’s sport (59) - Seek healthy sponsorship (14,55,77,91,92)   - E.g. Healthy food (i.e. Non-fast food) (91,92), non-food (92) - Encourage/negotiate with existing sponsors to implement healthy changes (19,64,65,73,76,92)   - E.g. offer healthy award option (76) - Designed to meet health and educational goals rather than commercial motives (19,52,103) - Unhealthy signage is replaced with healthy signage (19,87) - Restrict unhealthy branding (21,49,66,67,89,103)   - E.g. Unhealthy food logos on children’s uniforms (21)   - E.g. Sponsored/branding signage (23) - Health/recreation organizational logo can only be used with healthy foods (4) and is prohibited from being used on promotional materials with unhealthy foods (14,49,66,67,89) |
| Picks | N/A |
| Priming | Fundraising   - Healthy foods or non-food is used for fundraising (23, 122) - Fundraising with unhealthy food is discouraged or prohibited (79,87,101,106,107,108)   Rewards and incentives   - Healthy foods or non-food items are used for rewards/incentives (76) - Unhealthy foods are not used for rewards, incentives, vouchers (15,79,87), including for children and youth (15,76)   Promotional posters   - Health promotion messaging must be of equal weighting to messages from food and sugary drink sponsors, if the latter exists (61) - No unhealthy foods are used on promotional posters (87) |
| Place | No data. |
| Proximity | No data. |
| Policy | - Allowable sponsors are restricted (4,14,16,49,52,53,55,66,79,89,91,92,99,113) - Restricted unhealthy food promotion a condition of funding (16,99) - Negotiating healthier option within sponsorship agreement (92) - Sponsorship or marketing of unhealthy products or brands to children is prohibited (49,66,89) - Unhealthy options for fundraising and reward/incentives is prohibited (79) |
| People | - Staff (79) - Parents (21) - Food service (19) - Sports clubs (recreation facilities) (91) - Recreation organizations (49, 66, 67, 79, 89) - Sponsors (4, 14, 21, 22, 23, 26, 27, 28, 29, 30, 31, 35, 36, 37, 39, 41, 42, 43, 44, 49, 50, 51, 52, 53, 55, 58, 59, 61, 64, 65, 66, 67, 68, 69, 70, 76, 77, 79, 89, 91, 92, 98, 101, 102, 103, 106, 116, 124) - Municipalities (55,79) - Regional government (14, 21, 101, 106) - Facility/program funders (16, 27, 28, 29, 35, 36, 42, 43, 50, 69, 98, 99, 102, 106, 107, 108) - Non-government councils (15) |

References are document identification numbers (ID) as per Table 1 in Supplemental File 2.

**Table D. Indicators for Social Food Environment in Recreation and Sport Settings according to the HEERSS Framework**

| **HEERSS Component** | **Indicators for ‘Cook More Often’** | **Indicators for Enjoy Your Food, Including Culture and Food Traditions’** | **Indicators for ‘Eat Meals With Others’** |
| --- | --- | --- | --- |
| **Profile** | - Only monounsaturated or polyunsaturated oils are used for cooking (91, 93, 96) - Alternate preparation methods used (baking, broiling, grilling, steaming, roasting, toasting) instead of frying (7, 19, 96, 100, 89) - Recipes are followed to avoid the unnecessary addition of salt, sugar, fat (7, 19, 80) - Prepare using healthier ingredients: such as water, unsweetened milk, < 2% milk fat or fortified plant-based beverages (80) | - Have an awareness and understanding of the Maaori/Indigenous approach to growing, preparation and consumption of food (47) | No data. |
| **Portion** | No data. | No data. | No data. |
| **Pricing** | No data. | No data. | No data. |
| **Promotion** | No data. | - Make food labels available and visible so any potential religious conflicts can be seen (90) | No data. |
| **Picks** | No data. | - Food and beverages served and sold onsite reflect the cultural diversity of the community (23, 73) - Religious and cultural considerations will be taken into account when developing menus, etc. (47,103) | No data. |
| **Priming** | No data. |  | - Create a variety of seating options and social arrangements, recognizing that not all individuals will be (comfortable in a given configuration) (67) |
| **Place** | - Remove deep fryers from kitchens and source healthier alternative (23, 89, 96, 127) | No data. | No data. |
| **Proximity** | No data. | No data. | No data. |
| **Policy** | - Only monounsaturated or polyunsaturated oils are used for cooking (91) - Deep fryers are discouraged and other equipment is used to prepare unhealthy items (89) | - Have an awareness and understanding of the Maaori/Indigenous approach to growing, preparation and consumption of food (47) - Recognize the significance of cultures and religion on food (47) | No data. |
| **People** | Food retailers (23,89) | No data. | No data. |

References are document identification numbers (ID) as per Table 1 in Supplemental File 2.
